# A Comparative Evaluation of Current and Emerging Strategies for Almond Protein Extraction

**DOI:** 10.3390/molecules31122086

**Published:** 2026-06-14

**Authors:** Muhammad Adil Farooq, Jianmei Yu

**Affiliations:** 1 Institute of Food Science and Technology, Khwaja Fareed University of Engineering and Information Technology, Rahimyar Khan 64200, Pakistan; adil.farooq@kfueit.edu.pk; 2 Department of Family and Consumer Sciences, North Carolina A&T State University, 1601 East Market Street, Greensboro, NC 27411, USA

**Keywords:** almond protein, molecular structure, conventional extraction techniques, enzyme-assisted extraction techniques, physical and hybrid extraction techniques

## Abstract

Almonds (*Prunus dulcis*; family Rosaceae) contain 18–25% protein (dry weight). They are an important plant-based protein source in dairy alternatives and other functional foods. The hard and dense nature of almond kernels and the localization of proteins with lipid bodies in the cotyledons of almond seeds make it challenging to recover protein from the seed efficiently and preserve its function. Therefore, this review evaluates the influence of pretreatments, including blanching, grinding, and defatting, on almond protein recovery and functionality, and compares conventional and emerging technologies for almond protein. Traditional protein extraction techniques such as alkaline extraction–isoelectric precipitation (AE–IEP), aqueous extraction, and salt extraction provide moderate-to-high protein yields, but harsh processing conditions denature the proteins, decrease solubility, and cause functional properties to be lost. On the other hand, emerging protein extraction technologies (including enzyme-assisted aqueous extraction (EAE) ultrasound-assisted extraction (UAE), microwave-assisted extraction (MAE), high-pressure processing (HPP), and pulsed electric field (PEF) treatment) improve protein recovery, resulting in protein extract with superior functional properties and reduced allergenicity. However, their application in industry remain challenging. This review reveals that pretreatment approaches and conditions/parameters significantly influence protein extraction efficiency and the functional and structural properties of almonds, and that no single method is universally optimal. This review concludes that controlled enzymatic hydrolysis combined with physical pretreatment may be the best approach for producing high-value-added almond protein ingredients with specific techno-functional properties for use in plant-based beverages, hypoallergenic products, or nutraceuticals. More research is needed to develop an efficient, applicable, sustainable and eco-friendly almond protein extraction process, optimizing processing conditions to achieve high protein recovery while retaining desirable functional properties, and reduce operating costs.

## 1. Introduction

The global food industry is rapidly shifting toward plant-based proteins and dairy alternatives due to consumer interest in natural, healthy, lactose-free products, vegan diets, sustainability, and the diversity of protein sources. Plant-based milk alternatives are a significant and nutritious part of the retail food sector. There are numerous types of plant-based milk alternatives available to consumers today, including soy, oat, almond, coconut, and pea milks [1]. Almond beverages have been commercially successful primarily due to their neutral taste, pale appearance, and consumer appeal [2]. Almonds are among the three most commonly used raw materials in plant-based beverages. In addition, the nutritional characteristics of almond beverages vary widely based on the formulation and processing methods applied [3]. Around 40% of US households purchased almond beverages in 2024, down slightly from 44% in 2023, and almond milk holds the largest share, approximately 58%, of the US non-dairy milk market [4]. However, many commercial almond beverages do not contain adequate levels of protein compared to dairy milk and soy milk due to low protein solubility and a high water-to-almond ratio during processing. Thus, enhancing the efficiency of recovering and utilizing almond protein becomes even more important for developing new, high-value almond-based beverages, yogurt, desserts, spreads, and fortified foods.

Almond (*Prunus dulcis*) contains a number of nutrients, including considerable amounts of fat, protein, carbohydrate, fiber, vitamins, and minerals [5]. Protein in almond kernels can account for approximately 18–25% of dry weight [6]. Almond protein has a balanced amino acid profile that meets Food and Agriculture Organization (FAO) guidelines [6]. Furthermore, almond is a rich source of bioactive compounds, including phytosterols and polyphenols that contribute to various aspects of human health [7]. As a result of increasing consumer demand for sustainable, plant-based protein products, there has been an increase in research into new technologies for the efficient recovery of proteins from almonds while maintaining the nutrient quality and enhancing the functionality of the recovered almond proteins [8]. Compositionally, almond is primarily high in oil and has a moderate level of protein. The proteins associated with lipid bodies are localized in the cotyledons of almond seeds, creating challenges for extracting protein from this matrix.

The extraction and purification steps involved in plant-based protein production can cause proteins to aggregate and undergo other structural transformations that negatively affect their functional capabilities [9]. In the case of almonds, this problem is exacerbated by the fact that this kernel is not only a rich source of protein but also a very fatty material. The abundance of oils within almond kernels creates strong interactions between protein molecules and lipid droplets, helping stabilize emulsions formed during aqueous extraction of the protein. In addition, the strong affinity of the oil for the protein results in difficulty separating the oil from the protein-rich phase after extraction. Therefore, achieving efficient protein extraction from *P. dulcis* depends not only on maximizing yield but also on minimizing structural transformations that reduce the solubility, digestibility, and emulsifying/foaming capabilities of the resulting protein isolate [10]. de Souza et al. [11] and de Almeida et al. [12] demonstrated that the simultaneous extraction of oil and protein from almond kernels was significantly influenced by factors such as solid concentration, pH, extraction time, and enzyme usage. Their finding also demonstrates an additional major technological challenge in processing almonds into isolable protein: efficient extraction must be balanced against the effective removal of oil and insoluble solids from the protein-rich phase to obtain a high quality isolate with good functional performance in the desired application.

Kumar et al. [13] and Yang et al. [9] supported a paradigm shift in future plant-based protein processing toward less intensive and more selective extraction techniques that minimize damage to protein structure while reducing the amount of chemicals required. Since conventional solvent-defatted and alkali-extracted almond proteins tend to exhibit greater degrees of denatured aggregation than non-denaturing extraction methods (e.g., enzymatic extraction) and also generate more waste streams (e.g., acidulated wastewater), this need is especially critical for environmentally friendly and economically viable production strategies for almond-based foods.

Dias and De Moura Bell [14] found that the enzyme-assisted aqueous extraction method was highly effective for the simultaneous extraction of lipids and proteins from whole almond flour. This method offers several advantages: it does not require flammable solvents, allows simultaneous extraction of both components, and provides some control over the functionality of the extracted protein. In addition, recent investigations into sonication-, microwave-, and pressure-enhanced extraction techniques suggest that physical treatments can both enhance extraction efficiency and alter protein structures in beneficial ways, especially when used with enzymatic or aqueous processes.

Furthermore, the existing literature is scattered across studies that examine the composition of almond kernel protein, extraction methods, functional properties of almond kernel protein, beverage processing utilizing almond kernel protein, and utilization of the by-products generated during processing. However, there is currently little additional literature on almond kernel protein [6]. Likewise, broader reviews of plant protein extraction technologies discuss almond proteins only briefly in comparisons of plant matrices and therefore do not adequately address the unique lipid–protein co-extraction challenges associated with high-oil almond kernels [13,15,16]. Consequently, important questions remain insufficiently addressed, including how the structure of almond protein constrains extraction method selection, how different methods compare beyond extraction yield alone, and how process conditions influence application-specific functionality in beverages, yogurt alternatives, and emulsified foods. Therefore, the goal of this review was to evaluate both current and emerging methodologies for extracting almond protein. Initially, this review provides background on the chemical composition and structural features of almond proteins, as well as the effects of raw material preprocessing. Next, the review comparatively evaluates traditional extraction methodologies such as alkaline extraction–isoelectric precipitation, aqueous extraction, and salt extraction, followed by a discussion of newer approaches of enzyme-assisted, physically enhanced, and hybrid extraction methodologies. Finally, special emphasis is placed on how the process conditions affect the yield of protein extracted from almond flour, the separation of lipid from protein, the preservation of native protein structure(s), and functionality for specific uses. By integrating these elements, this review aims to identify efficient, sustainable approaches/strategies for producing almond protein ingredients for future plant-based food applications.

## 2. Composition and Structural Characteristics of Almond Proteins

### 2.1. Protein Content and Distribution Within Almond Kernels

**Figure 1:**
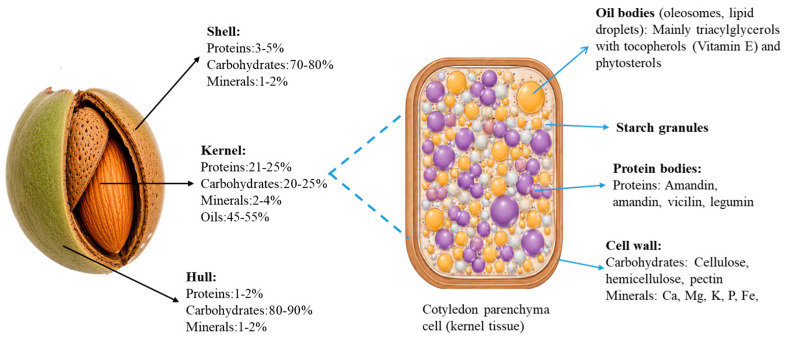
Organizational structure and compositionally distributed almond seed fruit and kernel. The diagram illustrates the outer layers (epicarp, mesocarp, and endocarp) and inner kernel as well as their respective contents of protein, carbohydrates, and minerals. An enlarged view of a cotyledon parenchyma cell illustrates an example of cellular organization that includes oil bodies (intracellular lipid containing triglyceride-rich structures), protein bodies (containing amandin and other types of storage proteins), and cell walls (hemicellulose, cellulose, and pectin). Original illustration created by the authors using BioRender/graphical design software (https://www.biorender.com/, accessed on 29 April 2026) and ChatGPT 5-assisted design tools based on information synthesized from the cited literature.

An almond seed consists of an embryo located at the center of the seed surrounded by a very thin, anatomically complex seed coat, as shown in Figure 1. The embryo itself contains two large cotyledons. Dourado et al. [17] demonstrated using fluorescence microscopy that both cotyledon cells contain numerous protein bodies and lipid bodies. These findings indicated that the almond kernel is a classic example of an oil storage tissue. Roncero et al. [18] subsequently confirmed the same microstructure in the cotyledons using light microscopy, scanning electron microscopy, and transmission electron microscopy. Trombetta et al. [19] visualized oleosomes throughout the seed using confocal laser microscopy, which they spatially correlated with protein bodies. These studies collectively suggest that almost all reserve protein in almond seeds is localized to the embryonic cotyledon tissue rather than primarily in the relatively thin outer tissues. Because most research on extracting and separating proteins from almond flour involves recovering protein from a lipid–protein composite matrix, understanding where reserve protein is located in an almond kernel is crucial for those interested in developing efficient almond protein extraction methods.

This spatial organization has important implications for almond protein extraction because proteins and lipids are closely associated within cotyledon cells and enclosed by cell-wall components such as cellulose, hemicellulose, and pectin. The plant cell wall acts as a diffusion barrier that restricts solvent penetration and protein release, while pectin-rich cell walls have been negatively correlated with protein extractability [20]. Furthermore, aqueous extraction liberates oleosin-stabilized oil bodies that form stable emulsions, particularly at neutral and alkaline pH, thereby complicating oil–protein separation during conventional extraction processes [19,21].

In addition to proteins and lipids, almond kernels contain significant amounts of low-molecular-weight compounds, particularly polyphenols, which are unevenly distributed within seed tissues. Most phenolic compounds, including flavonoids, phenolic acids, tannins, and proanthocyanidins, are concentrated in the brown seed coat (skin), and contribute substantially to the antioxidant capacity of almonds, whereas the cotyledons contain comparatively lower levels of these bioactive compounds [22,23].

These compounds can influence protein extraction behavior in several ways. Polyphenols readily interact with proteins through hydrogen bonding, hydrophobic interactions, or covalent associations in which polyphenols are oxidized. Such interactions may reduce protein solubility, promote aggregation, and decrease extraction efficiency during aqueous or alkaline processing [24]. In high-oil matrices such as almond, phenol–protein interactions may further complicate phase separation by stabilizing protein–lipid–polyphenol complexes during extraction. Consequently, preprocessing operations such as blanching and skin removal are commonly applied before protein extraction to reduce phenolic content, improve color, and enhance protein extractability. However, these treatments may also remove valuable antioxidant compounds and generate polyphenol-rich by-products with potential applications as functional food ingredients.

Therefore, the distribution and interactions of polyphenols should be considered alongside protein and lipid organization when designing almond protein extraction strategies, particularly for applications requiring high solubility, neutral flavor, and light-colored protein ingredients.

The total protein in sweet almond kernels generally falls within the range of 14–24 g per 100 g of fresh kernel [5], varying greatly with the specific variety, geographic origin, environmental conditions, and the nitrogen-to-protein conversion factor applied [18]. A factor of 5.18 was preferred over the typical value of 6.25 for almonds due to the fact that amandin, which constitutes approximately 80% of all proteins found in almonds, has an approximate nitrogen content of 19.3% [18]. Therefore, researchers should use extreme caution when comparing results directly among different studies based on their reported protein levels [5].

### 2.2. Major Almond Protein Fractions

Almond proteins are classified as a globulin–albumin-dominated system within the Osborne framework, with very small amounts of glutelins and prolamins [18]. Wolf and Sathe [25] refined that description by showing that water-extractable protein from defatted almond meal can be fractionated into 2S, 9S, 14S, and 19S proteins. They further identified amandin, a 14S fraction, accounting for approximately 65–70 percent of the extracted protein. Later Sathe et al. [26] found amandin to be the major storage protein in the almond kernel and confirmed it being a legumin-type globulin, making it the primary determinant of both physicochemical behavior and nutritional content of all almond protein ingredients. However, almond proteins are not limited to classical storage fractions, as the oleosome-associated extract contains oleosin, peroxygenase legumin, 7S globulin, and tonoplast intrinsic protein [19]. Zhang et al. [27] determined the molecular weights and isoelectric points of almond kernel proteins, broadly spanning the idea that the almond proteome is heterogeneous rather than chemically uniform. This matters because structural proteins such as oleosins help organize the lipid phase, while lower-abundance enzymatic or membrane-associated proteins may influence extraction selectivity, interfacial behavior, and even detection allergens. In that sense, the Osborne classification remains useful, but does not fully capture the spatial or functional complexity of proteins embedded in the almond oil-body–protein-body matrix.

### 2.3. Molecular Structure and Functional Properties of Almond Proteins

Amandin is made up of acidic polypeptides of approximately 42–46 kDa and basic polypeptides of approximately 20–22 kDa linked by disulfide bonds [26]. Amandin is an allergenic protein, named Pru du 6 by the WHO/IUIS Allergen Nomenclature Committee. It is a globulin; specifically a legumin-like hexamer with a molecular weight of approximately 360 kDa, indicating its multimeric structure stabilized by disulfide links. Furthermore, the present WHO/IUIS database lists seven allergenic proteins present in almond: Pru du 1, Pru du 3, Pru du 4, Pru du 5, Pru du 6, Pru du 8, and Pru du 10 [28]. Roncero et al. (2020) identified glutamic acid and aspartic acid as the two most abundant amino acids in almond protein [18]. However, Ahrens et al. [29] observed that sulfur-containing amino acids were the first limiting amino acids in almonds. Additionally, lysine and threonine became limiting when compared to additional stringent reference standards. Therefore, almond protein clearly provides good nutrition; however, it is not well balanced as a single protein source. Hence, it could benefit from supplementation with lysine-rich legumes.

A legumin-type 11S globulin, amandin possesses a hexameric structure stabilized by disulfide bonds and non-covalent interactions, similar to glycinin and pea legumin [26]. Under alkaline conditions, increased electrostatic repulsion promotes subunit dissociation and improves protein solubility and extraction yield, but excessive alkaline treatment may simultaneously induce denaturation, aggregation, amino acid modification, and loss of functional properties [30,31]. Conversely, near the isoelectric pH range (pH 3–5), almond proteins exhibit reduced solubility and increased aggregation, thereby facilitating isoelectric precipitation, but potentially compromising structural integrity and functionality [18,23]. Although Devnani et al. [32] obtained 70–80% solubility of almond protein isolate at pH 4 and 7, and they also found that acidifying the solution caused unfolding, increased the random-coil content, and produced lower-molecular-weight compounds via acidolysis [23]. They attributed these conformational changes to enhanced foamability, higher viscosities, and significant variations in gel microstructure post-heating. In addition, co-extracted oleosin-stabilized oil bodies exhibit low colloidal stability in the same pH range, further complicating phase separation during conventional extraction processes [21]. Thus, almond protein functionality is largely dependent upon protein structure, which is affected by pH and processing. Pru du 6 was reported as both a predominant storage protein and a major allergen that is thermally stable and causes serious adverse reactions after ingestion [33]. Zhang and Jin [34] further emphasized that the allergenic potential of almond proteins extended far beyond amandin. This indicates that the structural stability of almond proteins has a dual functionality: it protects functional properties during processing, but also maintains clinically relevant epitope structures contributing to the allergenicity of processed almonds [32].

## 3. Preprocessing of Almond Raw Materials

Almond kernels with longer shapes and greater amounts of skin and fiber will generally be damaged more easily. Hot-water blanching is typically used to separate the brown seed coat from the rest of the kernel before preparing flour. Fiskelments and Barrett [35] discovered that the removal of almond skins follows sigmoidal kinetics during blanching, and that blanching at 90–100 °C for 2–3 min is required to achieve effective skin peeling. The principal advantage of this step is twofold: it removes most of the lectins and a substantial fraction of the phenolic compounds concentrated in the seed coat, which otherwise bind nucleophilic protein groups and reduce protein extractability and digestibility, and it generates a cleaner, lighter-colored kernel suitable for ingredient applications [36,37]. These benefits carry three under-discussed limitations. First, blanching at 90–100 °C exceeds the denaturation temperature of native amandin and other 11S globulins (~80–85 °C for related legumin proteins), so the kernel entering the milling step is already partially denatured, even with brief blanching [6,26]. Second, studies of Pru du 6 (amandin) using sera from almond-allergic patients show that blanching and roasting do not reduce IgE-binding of the dominant 11S globulin bands. Hence, any expectation that preprocessing will reduce allergenicity in the final isolate is unsupported by the evidence [25,38]. Third, blanching generates a large quantity of by-product stream: blanch water and almond skins. The skins represent 4–8% of the shelled almond weight and carry the bulk of the kernel’s polyphenols and antioxidant activity into a waste fraction [18,22].

Due to the high lipid content of almond kernels, almond protein extraction requires preparing defatted, fine-particle almond flour [39]. Common strategies for removing lipids include mechanical pressing of blanched almond kernels or solvent extraction. Mechanical pressing includes cold pressing (at temperature 40–50 °C) and hot pressing (at 100–110 °C) by either expeller or hydraulic pressing machine. Cold pressing preserves native protein structures, bioactive compounds, and antioxidants and prevents the degradation of the oil [18]. However, it is less efficient, leaving a higher fat content in the resulting almond cake, which may continue to stabilize emulsions during subsequent aqueous extraction, thereby reducing protein separation efficiency. In contrast, hot pressing results in more complete oil extraction or less lipid residue in the almond cake, but elevated pressing temperatures can induce protein denaturation, aggregation, and structural modifications that negatively affect solubility and functionality [6,11]. Mechanical pressing of whole natural butte almonds at a temperature range of 73–80 °C resulted in the production of a cake with 16.25% oil and 37.20% protein according to de Souza et al. [11]. Typically, oil removal via screw pressing ranged from 70% to 90%, whereas oil recovery using solvent extraction exceeded 95%. Thus, mechanical pressing can be very attractive, since it allows the simultaneous production of a specialty oil and a protein-rich pressed cake without using organic solvents. The residual oil in the mechanically pressed almond cake is typically removed by additional solvent extraction using hexane to increase oil recovery and further enrich protein content [10,11]. Typically, oil removal via screw pressing ranged from 70% to 90%, whereas oil recovery using solvent extraction exceeded 95% [11].

Solvent extraction using hexane achieves substantially higher oil removal efficiency (>95%) and produces low-residual-fat meals that facilitate downstream protein extraction [12]. However, solvent exposure may alter protein structure through dehydration, aggregation, and disruption of oil-body organization [40,41]. Studies on oilseed and legume proteins further demonstrated that solvent-defatted meals often exhibit reduced protein nativity and altered functional properties compared with milder aqueous or mechanical extraction routes [13,41]. Consequently, although hexane defatting can improve protein extraction yield, its negative implications for sustainability and protein structural integrity have spurred growing interest in greener, less denaturing extraction approaches. In addition, hexane is a petroleum-derived flammable solvent, and hexane extraction presents important environmental, safety, and occupational health concerns.

After defatting, almond cakes are ground into flours of defined granularity. Grinding increases exposure surface areas of the almond particles and disrupts cell walls of the cotyledons, oil bodies, and protein-containing structures. This fact was reinforced by Hallstrom et al. [42], who demonstrated that decreasing the particle size of almond flour from flour to butter or paste increases oil recovery to up to 96% and significantly improves protein recovery during enzyme-assisted aqueous extraction to nearly 92%. However, the same research also indicated that increasing particle size would lead to increased rupture of oil bodies, cellular disruption, and protein aggregation in the more finely ground products. Therefore, grinding can enhance mass transfer, but alter the original arrangement of almond matrix proteins and lipids. Therefore, the effects of particle size on protein recovery also have two aspects, and the degree of grinding has to be optimized to achieve maximal protein extraction.

## 4. Conventional Extraction Methods for Almond Proteins

The traditional extraction methods are based on solubility variation of proteins with pH and ionic strength. Using this principle, the majority of plant seed storage proteins (globulins and albumins) can be isolated. Most commonly used techniques for isolating proteins from almonds include aqueous extraction and/or salt extraction or alkaline solubilization followed by subsequent isoelectric precipitation. They can be performed at a low cost with relatively high capacity and ease of operation [8].

### 4.1. Alkaline Extraction–Isoelectric Precipitation

Alkaline extraction–isoelectric precipitation (AE–IEP) has been one of the primary methods for producing protein isolates from almonds (*Prunus dulcis*) because it represents an eco-friendly and technologically accessible method for the extraction of high-purity almond proteins. During the alkaline extraction step, defatted almond flour is mixed with water and the pH is adjusted to 9.0–11.0 using sodium hydroxide. This increases the solubility of the major proteins found in almonds, amandin and 7S vicillin-like proteins. The remaining insoluble cell-wall polysaccharides, proteins, and lipids are separated from the solution by centrifugal separation [10]. The resultant protein-rich solution is then acidified to pH 4.5–5.0 (the isoelectric point of amandin), causing the proteins to flocculate and precipitate from the solution due to electrostatic attraction and protein–protein interactions that occur through charge neutralization and subsequent aggregation of the proteins in the solution [43]. Once this occurs, the precipitated protein mixture is again separated by centrifugation or filtration followed by washing and neutralization of the solution. Finally, after freeze-drying of the precipitated protein mixture, high-quality almond protein isolates containing 85–92% protein are typically produced, indicating that AE–IEP is effective at extracting almond proteins from a highly complex matrix [44]. Significant drawbacks of this method include low protein yield and high environmental impacts. Typically, this method extracts only about 40–50% of the available protein in almond flour. The harsh pH conditions can slightly denature the proteins, which may negatively alter their functional properties (e.g., foaming, gelling). It also generates a substantial amount of wastewater.

Studies optimizing various parameters of the AE–IEP process have shown that the extraction pH, temperature, solid/liquid ratio, and ionic strength were the four factors that had the greatest impact on both the yield and quality of almond proteins recovered using AE–IEP. Increasing the extraction pH from 9.0 to 10.5 enhanced the extent of protein solubilization; however, it also resulted in greater co-solubilization of phenolics. Thus, an optimal pH for extracting almond proteins was determined to be 9.5–10.0 [45]. Temperatures of 40–55 °C enhanced protein recovery primarily by increasing molecular mobility within the system and reducing the apparent viscosity of slurries formed during processing; however, temperatures > 55 °C may cause irreversible denaturation of amandin before its isolation [43]. The solid/liquid ratio is another factor that impacts both the extent of protein solubilization and downstream processing efficiency. The most commonly reported solids-to-liquid ratio is 1:10 to 1:15 (*w*/*v*) [14]. Reducing agents such as sodium sulfite may be used to break the intermolecular disulfide bonds linking individual amandin hexamer units, thereby enhancing both the solubilization and precipitation yields of amandin [32,46].

The functional properties exhibited by AE–IEP-derived almond protein isolates depend upon the combination of intrinsic molecular structure(s) of amandin and extrinsic modification(s) imposed on those structures by exposure to alkaline and acidic solutions. Solubility of AE–IEP-derived almond protein is relatively low (approximately 30–55%) at neutral pH, resulting from partial denaturation during extraction and precipitation processes. However, solubility increases substantially at pH 8.0 or higher, allowing AE–IEP-derived almond proteins to be utilized in food systems with elevated pH levels (e.g., beverages and formulations containing sodium bicarbonate) [11,47,48]. Another functional property is gelation behavior. AE–IEP almond proteins exhibit excellent gelation characteristics at a concentration of 10% (*w*/*v*) or higher. Heating induces thermal denaturation, enabling disulfide bonding and the formation of networks capable of producing gels with suitable textural properties for use as structuring components in plant-based foods [49]. From a nutritional perspective, AE–IEP-derived almond proteins exhibit favorable profiles of essential amino acids (leucine, arginine, glutamic acid), although protein digestibility-corrected amino acid score (PDCAAS) values are diminished by phenolic cross-linking and residual trypsin inhibitory activity. Consequently, it is critical to optimize AE–IEP processes to simultaneously maximize yield, purity, and nutritional quality [50,51]. Green chemistry techniques, which include subcritical water extraction of almond by-products, are emerging means to recover polyphenol-rich bioactives from almond skin [50]. Researchers continue to explore optimal conditions of the conventional AE–IEP methods to enhance both the functional and nutritional properties of almond proteins [51,52].

### 4.2. Aqueous Extraction Process

Aqueous extraction processes (AEPs) have been recognized as environmentally friendly alternatives to traditional hexane-based extraction methods for almond proteins. In AEP, water is used as an extraction medium [53]. AEP commonly involves dispersing defatted or full fat almond flour in an aqueous solution under alkaline conditions (pH 8.0–9.5) at elevated temperature (40–60 °C) and a controlled solid-to-liquid ratio, followed by centrifugation to recover protein-rich skim fractions. Compared to AE–IEP, the protein extraction efficiency of AEP is lower. A study reported approximately 56% protein extraction yield from full-fat almond flour when treated at pH 9.0 and 50 °C by conventional AEP. Higher recovery of amandin was obtained under alkaline conditions compared to the other protein fractions [14]. Studies optimizing AEP have found that low solid-to-liquid ratios (1:10–1:13) and longer extraction times (1–2 h) can increase protein yield by approximately 70% [11]. Enzyme-assisted AEP (EAEP) involves adding proteases (0.5–0.85%) to enhance the extraction of both oil and proteins. EAEP has resulted in protein yields of approximately 75% in less processing time than AEP [14]. The proteolytic breakdown produces smaller peptides, creates a more disordered secondary protein structure, and reduces surface hydrophobicity of the extracted proteins [54]. Furthermore, the cellular matrix of almond cotyledons is disrupted by the alkaline conditions, which facilitate the release of intracellular protein and lipids.

The extraction method greatly affects the technological functions of almond proteins. For example, EAEP-extracted proteins show greater solubility (47% vs. 23% at pH = 5.0), emulsification capacity (492 g oil/g protein vs. 402 g oil/g protein), emulsion activity index (35 m^2^/g vs. 17 m^2^/g), and foaming capacity (23% vs. 11%) compared to AEP–IPE proteins, especially near their isoelectric points [14]. Water-holding capacity also increased from 2.4 to 2.7 g water/g protein after enzymatic treatment. However, AEP often leads to lower protein recovery than AEP–IPE due to the lower extraction pH (up to 9.0) at which the solubility of plant globulins is low.

### 4.3. Salt Extraction

Salt extraction is a traditional method of isolating the storage proteins from seeds. It uses the fact that globular storage proteins dissolve more readily in an aqueous salt solution. This is especially true for the major storage protein amandin. The basic principle of salt extraction is based on the Hofmeister and Debye-Hückel theories. According to these theories, dissolved salts such as sodium chloride (NaCl) or sodium phosphate in buffers can act as counterions, neutralizing the charge on the surfaces of protein molecules [55]. As a result, there will be less electrostatic repulsion between charged amino acids. Additionally, as the charge on the surface decreases, there will be greater hydration of the hydrophobic regions of the protein molecule. Both factors will cause an increase in the amount of protein that dissolves in the salt solution. This phenomenon is referred as “salting-in.” A comparison to alkaline extraction–isoelectric precipitation (AE–IEP) shows that salt extraction occurs at near-neutral pH (6.5–8.0), which is very close to the pH found within seed storage tissue. Thus, salt extraction minimizes acid- or base-induced denaturation, racemization, and lysinoalanine formation during the extraction process. Therefore, salt extraction preserves much of the native quaternary structure of amandin along with many of its functional properties [56].

Almond proteins obtained by salt extraction exhibit characteristics indicative of structural integrity. Electrophoretic mobility data from non-denaturing polyacrylamide gel electrophoresis (native PAGE) indicate that these proteins have nearly normal electrophoretic mobilities [25]. Circular dichroism spectroscopy provides evidence that the secondary structures of these proteins have largely remained intact throughout the extraction procedure [57,58]. Thus, salt-extracted almond proteins differ significantly from those extracted using AE–IEP procedures.

As with all purification techniques involving extraction medium and separation of desired components from unwanted ones, optimal conditions exist to maximize protein yields and purities while minimizing the co-extraction of lipids, phenolics, and non-protein nitrogen. Among the several parameters that influence the efficiency and selectivity of salt extraction for almonds, two primary parameters are salt concentration and salt type. Solutions of sodium chloride (NaCl) over a range of concentrations (0.1–0.5 M) are most often utilized for extracting the globulins from almonds, and maximal solubilization of amandin is typically achieved at 0.5 M NaCl. Concentrations above 0.5 M NaCl may lead to salting-out effects that reduce protein extractability [26]. Combinations of NaCl and phosphate buffers (pH 7.0–7.5; 0.05–0.1 M) are often used to maintain pH stability and improve consistency among multiple extractions. Phosphates also chelate divalent cations that could otherwise contribute to protein aggregation or co-precipitation with phytate–protein complexes. Conditions related to agitation during extraction (e.g., speed and period of agitation) affect how thoroughly protein is solubilized. Diminishing protein recovery may be observed during agitation periods longer than 2 h under typical conditions [59]. Protein recoveries from extracts are usually performed by dialyzing against distilled water or low-ionic-strength buffers in order to decrease salt concentrations gradually and cause controlled precipitation of globulin near their respective isoelectric points (~5.0–5.5 pI). Alternatively, ammonium sulfate can be added to selectively precipitate protein subfractions at particular saturation levels ranging from 20% to 80%. Membrane-based salt removal using ultrafiltration/diafiltration processes may represent an advantageous, more scalable, and more efficient replacement for traditional dialysis techniques. As both concentration and desalination occur in a single processing step, processing time decreases from 12–24 h (dialysis) to 2–4 h [60].

Salt extraction produces almond protein isolates with superior retention of their native structures and functions compared with protein isolates produced by AE–IEP or alkali extraction. This superiority stems primarily from the mild ionic nature of the salt extraction process, which occurs at near-neutral pH throughout the process. Solubility of the isolated amandin, resulting from salt-extraction, was reported to be notably greater than AE–IEP isolates over a pH range of 6.0–9.0. For example, the solubility of amandin from salt-extraction was 80–95% at pH 8.0, in contrast to only 40–65% extracted by AE–IEP under comparable conditions [61]. Salt-extracted almond protein has higher emulsifying activity index (EAI) and emulsion stability index (ESI) values than AE–IEP isolates, owing to the preservation of the native organization of the amphipathic domains within amandin, which enables rapid film formation at oil–water interfaces. These characteristics make salt-extracted almond protein particularly desirable for use as a protein substitute in plant-based dairy beverages, salad dressings, and infant formula analogues [26].

Compared to standard alkaline extraction methods, salt extraction has several disadvantages. First, although its protein extractability (55–75%) is higher than AEP alone, it is lower than AE–IEP (75–88%) under optimal conditions. The low recovery observed in salt extraction results from the inability to extract a portion of the albumin- and glutelin-type proteins present in almonds under moderate ionic strength conditions [62]. Second, salt extraction needs costly and complex downstream processing. High salt concentrations are needed to solubilize the proteins, which must then be removed by energy-intensive, expensive downstream processes like ultrafiltration, diafiltration, or a prolonged dialysis desalination process to achieve food-grade purity [58]. Third, using high-concentration salt buffers generates large volumes of saline wastewater, and its disposal is an expensive, environmentally unfriendly process. In addition, chlorogenic acid is the primary phenolic compound in almonds and poses a common quality-control challenge. When extracted at near-neutral pH, chlorogenic acid forms ion pairs and associates with protein, producing either non-covalent associations or oxidative covalent cross-linking with amandin. These interactions are detrimental to the nutritional functionality of the product, as they contribute to reduced digestibility, greenish/brown coloration, and unpleasant astringency/flavor defects. Treatment of extracts with activated carbon or acid washes of the precipitates can help minimize polyphenol content, but needs to be conducted carefully to limit loss of protein [26]. Further, due to the conservation of native structure through salt extraction, the allergenic potential of salt-extracted amandin remains relatively unchanged. Therefore, allergens must be clearly labeled on all derivative foods in regulated food markets where allergens are strictly defined [25]. Overall, salt extraction is the preferred means of producing intact, native almond protein isolates suitable for detailed structural analysis or high-end application development on a laboratory scale. However, there are opportunities for future commercialization by developing more cost-effective continuous membrane desalting technologies and pretreatment technologies for defatting almond meal to enhance scalability advantages compared to AE–IEP and aqueous extraction processes.

It is clear that each conventional extraction method has its pros and cons and can be used for specific cases. Table 1 presents the findings of almond protein extraction studies using different conventional protein extraction methods. Table 2 compares the advantages and disadvantages of these conventional methods for almond protein extraction.

## 5. Enzyme-Assisted Extraction Strategies

Enzyme-assisted extraction (EAE) of plant proteins is a green, sustainable, and efficient technique that uses enzymes such as cell wall-degrading enzymes (carbohydrases) and proteases to break down plant cell walls and partially hydrolyze proteins, thereby increasing extraction yields under mild conditions.

### 5.1. Cell Wall-Degrading Enzyme-Assisted Protein Extraction

One major issue with almond protein extraction is the presence of the rigid plant cell wall, which consists mainly of cellulose, hemicellulose, and pectin, and forms a complex polysaccharide matrix that severely restricts solvent access to the intracellular protein bodies [67]. Using cell wall-degrading enzymes (CWDEs), including cellulases, pectinases, hemicellulases, and xylanases, offers a relatively environmentally friendly way to overcome this structural barrier by breaking down the individual component(s) of the cell walls under relatively mild process conditions [68,69].

CWDEs cleave the specific glycosidic bonds in the cell wall matrix. Cellulases cleave the cellulose backbone. Pectinases cleave the pectin network that holds neighboring cells together. Hemicellulases cleave the hemicellulose cross-linking sites. Taken together, they weaken the overall structural integrity of the cell wall and allow the release of the entrapped proteins [70,71]. Multi-enzyme commercial preparations such as Viscozyme^®^ L (arabanes, β-glucanase, cellulase, hemicellulase, and xylanase) and Celluclast^®^ have been used to improve protein extraction yields from various plant matrices. In peanuts and rapeseed, cell wall-degrading enzymes ( Viscozyme^®^ L., cellulase, hemicellulase, pectinase, pectinase-rich preparations) increase protein extraction by 1.4–1.7-fold without hydrolyzing protein compared to conventional methods [72,73]. Studies on almond cake have demonstrated that enzyme-assisted aqueous extraction processes using CWDEs increase protein extraction yields to 70% compared with 48% by conventional aqueous extraction. Additionally, EAE process facilitates oil recovery [11,14]. The amount of enzyme added, incubation time, pH, and temperature are important variables affecting the degree of cell-wall breakage and protein release [74].

EAE can modify the techno-functional characteristics of the extracted protein. Treatment with CWDEs followed by protease treatment yields smaller peptides with higher zeta-potential values and lower surface hydrophobicity. These modified proteins show dramatically improved solubilization, reaching 80–90% under optimal conditions compared with native proteins [11]. Their emulsification capacity around the isoelectric point has greatly improved. For example, enzyme-treated proteins had an emulsification activity index (EAI) of 35 m^2^/g compared with 17 m^2^/g for unmodified almond proteins [14]. Likewise, the foam capacity of enzyme-treated almond proteins was greater than that of untreated proteins, from 11% to 23% at pH 5.0. Water absorption capacity (WAC) also increased from 2.4 g water per gram protein to 2.7 g water per gram protein because more polar groups were exposed due to structural unfolding [54]. However, over-hydrolysis can reduce emulsifying capacity and foam stability, since very small peptides do not effectively stabilize surfaces [68].

Enzymatic treatment of almond proteins also induces substantial changes in their physical/chemical properties. Protein secondary structures become more disordered after enzymatic treatment—increasing random coil content and decreasing α-helices and β-turns [75]. As a result of this structural rearrangement, thermostability decreases while in vitro protein digestibility increases from 79.1% to 88.5% [41]. Moreover, modest enzymatic hydrolysis can decrease immunogenicity by ~75%, thereby enabling the production of hypoallergenic almond-based foods [54]. Enzyme-treated almond proteins also exhibit substantially greater antioxidant capacity and α-glucosidase inhibitory activity (~98%), indicating potential as nutraceuticals for preventing or treating metabolic disorders [45].

These benefits are bound by limitations that the existing almond literature rarely consolidates. First, commercial CWDEs only partially degrade the plant cell wall: many proteins remain trapped within incompletely lysed cells even at high enzyme doses, imposing a yield ceiling well below theoretical value [76,77]. The 70% protein recovery on almond cake reflects this ceiling, rather than a process maximum that further optimization can readily exceed [11,14]. Second, CWDEs such as Viscozyme^®^ L contain trace protease activity that can initiate uncontrolled hydrolysis of the released protein [78]. Third, the gains in protein solubility, EAI, and foam capacity follow a bell-shaped relationship with the degree of hydrolysis (DH): functional properties peak at low–moderate DH and then collapse as further proteolysis produces peptides too short to form a cohesive interfacial film [79]. Over-hydrolysis is therefore a real industrial risk, particularly because CWDE preparations with protease contamination can drift the optimal extraction condition without operator intervention. Fourth, although yield gains and oil co-recovery are operationally attractive, CWDE is widely flagged across cross-cutting reviews as slow, expensive to run, difficult to scale, prone to uneven yields, and energy-intensive—caveats rarely acknowledged in process-development reports on almond [75,80]. Enzyme cost is the principal scale-up barrier, with EAE often justified economically only for high-value products. Immobilized enzyme systems and enzyme recycling can mitigate this cost, but have not been integrated into reported almond EAEP processes [75,80]. CWDEs are therefore a powerful tool when the target product is a near-isoelectric beverage or emulsion ingredient and when DH is held within a narrow operating window, but the method requires tighter control of pretreatment, dose, and endpoint than conventional alkaline extraction does.

In summary, CWDEs offer an environmentally friendly means to enhance the yields of extracted almond proteins while improving both techno-functional and physical/chemical properties. Optimization of the type of enzyme used, its concentration, and processing conditions is required to extract maximum amounts of functional protein without causing excessive hydrolysis that could potentially negatively impact the desired functionalities of the extracted proteins.

### 5.2. Protease-Assisted Protein Recovery

Recently, protease-assisted extraction (PAE) technologies have emerged as environmentally friendly methods for selectively cleaving peptide bonds, allowing the fragmentation of large proteins into smaller peptides that can be extracted more easily from the cellular matrix [8,75]. The most commonly used commercial proteases for aqueous extraction processes of almond proteins include Alcalase^®^, neutral proteases derived from *Bacillus subtilis* and *B. amyloliquefaciens*, and Flavourzyme^®^ [14]. For example, 0.5% (*w*/*w*) enzyme is typically added to an aqueous slurry of almond flour or cake with a solid-to-liquid ratio of 1:10 at pH 9.0 and 50 °C, for 60 min. Under these conditions, PAE achieved an average of 70–75% protein recovery, whereas AE yielded approximately 67–70% protein recovery [12,47]. Treatment of almond protein isolates with Alcalase^®^ resulted in a degree of hydrolysis (DH) value of 10.95% after pretreating with ultrasonic waves, which was found to increase protein recoveries from de-oiled almond meal [81].

The use of proteases to recover almond proteins profoundly alters their techno-functional properties. Hydrolysis to a DH of 7% significantly enhanced the solubility of the protein, especially around its isoelectric point, where it increased from 23% to 47% at pH 5.0 [14]. At pilot-plant scale, protease treatment increased total solubility to 95.3% at pH 9.0 by altering the zeta potential and reducing the surface hydrophobicity [12].

Protease-assisted extraction has profound impacts on the functional properties of plant protein depending on the type of protease and DH, which can be managed by selecting a proper enzyme and controlling hydrolysis conditions including protease-to-substrate ratio concentration, hydrolysis time, pH, and temperature. The emulsifying capacity of treated almond protein increased from 402 to 492 g oil/g protein and the emulsifying activity doubled from 17 to 35 m^2^/g at pH 5.0. Similarly, the foam capacity rose from 11% to 23%. The maximum values for both solubility and foam expansion were obtained by treating an almond protein isolate with Alcalase (2.4 U/g) for 60 min [79]. Additionally, water absorption capacity also improved from 2.4 to 2.7 g H_2_O/g protein after protease treatment [14]. Nevertheless, over-hydrolysis produces a lot of very small peptides that will likely negatively affect the long-term stability of emulsions and foams, underlining the importance of controlled and moderate proteolysis [12,79]. The effects of DH on the functional properties of plant protein follow a bell-shaped curve. The solubility, emulsifying activity, and foam capacity are typically reach their peaks at low–moderate DH (5–10% for legumin-type globulins), and then collapse as further proteolysis produces peptides too short to anchor a stable interfacial film [82]. For almond, the published data define only the ascending side of this curve. The optimum and descending side are poorly characterized, and uncontrolled hydrolysis is consequently a real industrial risk. Emulsion and foam stability collapse earlier and more sharply than the capacity metrics because stability depends on interfacial film cohesion rather than initial coverage [12,79]. In addition, hydrolysates with DH > 5% cannot form heat-induced gel [79]. Extensive protease treatment destroys the hexameric quaternary structure of amandin and eliminates thermal stability, resulting in too many smaller peptides, which lack sufficient molecular size and structural flexibility/strength and thus are unable to create the dense, viscoelastic interfacial films required to stabilize emulsions and foams [80].

Protease-assisted extraction induces profound changes in the physical and chemical properties of almond proteins. Specifically, the application of proteases leads to the unfolding of secondary structure, with a greater proportion of random coils and lower percentages of α-helices and β-sheets, resulting in a loss of thermal stability [50]. Protease hydrolysis preferentially degraded the amandin α chain, whilst the β chain remained relatively unaffected, creating a specific peptide profile that influenced downstream functional properties [54].

Moreover, PAE increased the in vitro protein digestibility of almond protein from 79.1% to 88.5%, since smaller fragments are more susceptible to digestive enzymes [54]. More importantly, protease treatment reduced immunoreactivity of almond protein extract by around 75%, due to the modification or destruction of conformational and sequential epitopes on allergenically relevant proteins such as prunin 1 and 2 [54]. Finally, some generated peptides exhibited strong antioxidant activities and showed almost complete inhibition against α glucosidase (>98%) suggesting that they could potentially be useful for controlling metabolic disorders [53]. Furthermore, ultrasound and microwave pretreatments before applying Alcalase^®^ hydrolysis further increased the antioxidant activity and TCA-solubility of the corresponding hydrolysate [81].

Therefore, protease-assisted protein recovery is a valid, economically viable, and environmentally sustainable method for recovering almond proteins and improving their technological and biochemical properties. It is essential to perform controlled, moderate proteolysis to optimize solubilization, emulsification and digestibility without affecting the interfacial stabilization, thereby extending the possible uses of almond proteins in functional food products and hypoallergenic formulations.

EAEP nevertheless inherits the limitations identified above for CWDE and protease-assisted routes, in some respects more acutely. The three-fraction split is operationally attractive, but the cream-emulsion fraction retains a significant share of the protein adsorbed at the oleosin-stabilized oil–water interface, requiring a downstream demulsification step that has not been resolved at scale [10,12]. The bell-shaped DH–functionality relationship applies to the skim fraction in the same way [45,78], so EAEPs must hold protease activity within the optimum window despite the additional variability introduced by carbohydrase action on the matrix.

In conclusion, aqueous and enzyme-assisted aqueous extraction provide green ways for recovering high-quality almond proteins with better technological functionalities and physical/chemical properties. A low degree of enzymatic hydrolysis mainly enhances solubility, emulsifying capacity, foaming capacity, digestibility, and bioactivity of almond proteins, thereby expanding possibilities for applying almond proteins in functional food and nutraceutical formulations.

## 6. Emerging Physical Extraction Technologies

Emerging physical technologies such ultrasound-assisted extraction (UAE), microwave-assisted extraction (MAE), high-pressure processing (HPP), and pulsed electric field (PEF) treatment offer distinct mechanical advantages in disrupting the cellulose pectin cell wall matrix of almond cotyledon cells while maintaining the peptide backbone of amandin. UAE, MAE, HPP, and PEF treatments selectively target non-covalent interactions (hydrogen bonding, hydrophobic association, and electrostatic attraction) in order to produce a controlled unfolding of proteins, a disassociation of aggregates, and a reduction in particle size, thus creating improved techno-functional properties (solubility, emulsion-forming capability, foam-producing capability) without generating chemical modifications. Additionally, several of the abovementioned methods enable the simultaneous extraction of valuable bioactive compounds (polyphenols and condensed tannins) and produce an immunologically reduced almond by disrupting conformational epitopes. The next sections will discuss each of the above treatments in terms of their effect on almond protein recovery, structural modification, and function.

### 6.1. Ultrasound-Assisted Extraction

**Figure 2:**
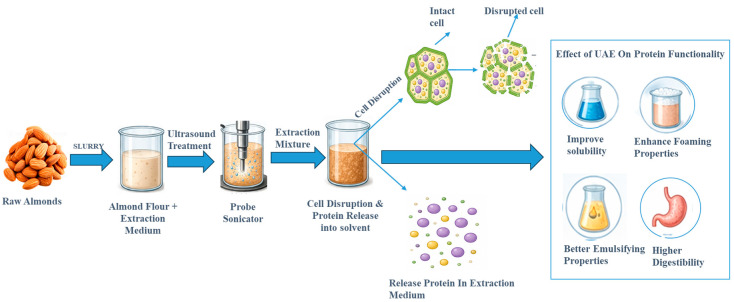
Schematic illustration of ultrasound-assisted extraction (UAE) of almond proteins and its impact on protein functionality. This figure was created by the authors using BioRender/graphical design software and an AI-assisted design tool (Canva) based on information synthesized from the cited literature.

Ultrasound-assisted extraction (UAE) has attracted considerable interest recently as an eco-friendly and efficient method for recovering proteins from plant materials. UAE uses high-frequency sound waves (typically 20–100 kHz) to generate microscopic cavitation bubbles in a liquids. The rapid formation and violent collapse of these bubbles create immense mechanical shear forces and localized high temperatures, which break cellular structures and accelerate protein release (Figure 2). During sonication, cavitation-induced microjets and turbulence will rupture cell walls and break up larger protein complexes allowing the intracellular proteins to be released into the extraction solution. Thus, it can both increase protein yield during almond protein extraction and modify the structure and functionality of the extracted proteins without using chemical solvents [75,83].

The sonication parameters affecting protein extraction efficiency and protein structural modification include ultrasonic power level, treatment time, ultrasonic frequency, and temperature. All these factors need to be optimized to prevent excessive denaturation or aggregation of the protein. For the UAE of almond proteins, aqueous suspensions or slurries containing proteins are treated with various levels of ultrasonic power (200–600 W) and time (15–30 min) and usually with a frequency range of 20–25 kHz. Sari et al. [81] found that treating defatted almond meal with ultrasound before enzymatic hydrolysis using Alcalase increased protein recovery by 116% and achieved the highest degree of hydrolysis at 10.95% after 50 min of sonication. Sari et al. [83] showed that when high-pressure microfluidization was used with sonication, it improved the structural disruption of the almond meal proteins even further.

Sonification greatly enhances the technological function properties of almond protein isolate (API). A study found that water solubility increased from 60.4% in control samples to 95.2% following sonication at 600 W for 15 min. This was attributed to the breaking up of large protein aggregates into smaller, more water-soluble particles. The emulsifying activity index (EAI) value was elevated from 12.8 m^2^/g to a maximum of 37.4 m^2^/g at 400 W for 30 min, whereas emulsifying stability index (ESI) values were also considerably improved. Both foam formation and foam stability were significantly improved in all sonicated samples [50]. The use of an API modified by sonication resulted in excellent Pickering emulsion stabilization properties with respect to their application in meat products. Yang et al. [84] have shown that at a sonication power level of 300 W, API exhibited an EAI of 87.65 m^2^/g and foaming stability of 90.93%. The physical stability of oil-in-water emulsions created with API as Pickering emulsion stabilizers was significantly enhanced through sonication treatment, as evidenced by smaller particles of the emulsion droplets stabilized by API, higher critical osmotic pressure, and greater protein interfacial adsorption [84].

FTIR analysis revealed significant physicochemical alterations in almond proteins caused by sonication. There is evidence that sonication causes protein unfolding, as evidenced by a significant reduction in α-helices and β-turns and a simultaneous increase in random coils [75,85]. The number of surface-free sulfhydryl groups is significantly increased due to the increased exposure of previously buried thiol groups. Intrinsic fluorescence intensity is reduced at higher power levels and longer sonication times suggesting structural alteration to the micro environment surrounding aromatic amino acids. The mean particle diameters (D43 and D32) are significantly reduced from approximately 89.77 µm and 10.6 µm to 20.01 µm and 4.97 µm, respectively, after sonication treatment at 400 W for 30 min resulting from the breakdown of protein aggregates [75]. Ultrasound pretreatment has also been shown to enhance the antioxidant activity of subsequent Alcalase hydrolysates, with ORAC values significantly greater than their untreated controls, indicating that small bioactive peptides are generated and the TCA-solubility index increases [81]. Vanga et al. [86] have shown that pulsed ultrasound treatment alters the secondary structure and enhances in vitro digestibility of almond milk proteins.

Ultrasound-assisted alkaline extraction has recently been evaluated for its ability to recover almond protein. The application of ultrasonic energy has resulted in higher protein recovery efficiencies than mechanical agitation alone. The observed yield enhancement is attributed to ultrasonic energy-induced disruption of cells and release of proteins from intact cells [87]. Although emulsifying activities and stabilities are relatively good, post-extraction ultrasound treatment can significantly improve emulsifying capacities by facilitating the unfolding of amandin and exposing previously occluded hydrophobic domains [87].

### 6.2. Microwave-Assisted Extraction

**Figure 3:**
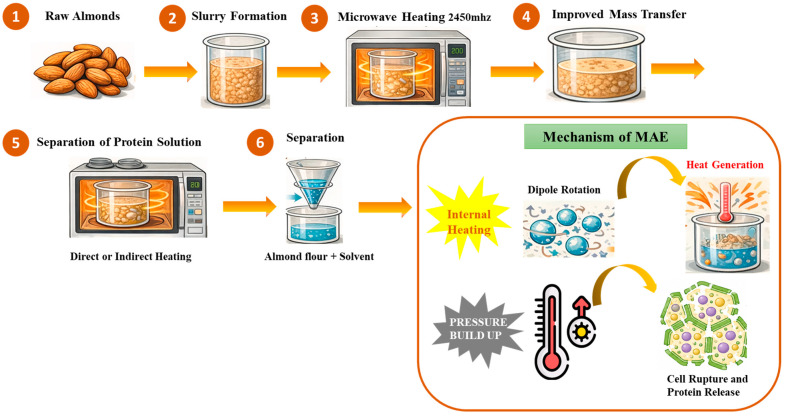
Mechanistic illustration of microwave-assisted extraction (MAE) and its role in cell rupture and protein release. Red arrows indicate heat generation and temperature rise, whereas yellow arrows denote the sequence of mechanistic events leading to enhanced extraction efficiency. Original illustration created by the authors using BioRender/graphical design software and an AI-assisted design tool (Canva) based on information synthesized from the cited literature.

Microwave-assisted extraction (MAE) is an emerging quick, efficient, and green method for the recovery of bioactive compounds and proteins from plant-based materials. Different from traditional heating methods, microwaves are capable of delivering electromagnetic energy to the polar molecules present in the plant matrix, resulting in homogeneous volumetric heating that causes internal pressure build-up inside the cells to exceed the mechanical strength of the cell walls, leading to cell rupturing and increasing mass transfer of intracellular contents into the extraction solvent [67,74]. Since this process is particularly effective for quickly disrupting the rigid cell wall barriers of almonds, it also minimizes the need for extended thermal treatments that may result in irreversible denaturation of sensitive protein fractions [51,81]. Figure 3 demonstrates the process and mechanism of MAE of almond protein.

The MAE of almond proteins typically involves dispersing defatted almond meal or flour in aqueous or buffer solutions, followed by microwave irradiation at controlled power levels (typically 160–800 W) for relatively short periods (45–225 s). For example, using MAE on defatted almond meal after microwave pretreatment at 160 W for 45 s prior to Alcalase hydrolysis resulted in an approximately 1.18-fold increase in protein yield and a degree of hydrolysis (DH) of 8.87%, whereas the DH of untreated controls was about 5.76% [81]. Rapid dielectric heating caused by microwave unfolded protein structures, making accessible previously inaccessible cleavage sites such that enzymatic hydrolysis occurred more rapidly and generated lower-molecular-weight peptides that had higher biological activity [88,89]. In addition, a recent comparative study of MAE and UAE on okara (the solid residue of almond from almond milk production) indicated that 400 W was the optimal microwave power for maximum extraction of bioactive compounds from okara, above which thermal degradation became apparent [90]. As excessive microwave power can lead to protein aggregation and/or degradation of heat labile amino acids, temperature control is important during MAE [68].

MAE also significantly affects the technological and functional characteristics of almond proteins. Rapid structural unfolding caused by microwave irradiation increases protein solubility by exposing their hydrophilic residues and reducing particle size, a characteristic consistent with other plant protein systems [8,83]. The MAE-modified almond protein hydrolysate exhibits a greater trichloroacetic acid (TCA)-solubility index, indicative of the formation of smaller soluble peptides [81]. Enhanced emulsifying activity was observed with MAE-modified proteins due to the exposure of hydrophobic patches through partial unfolding, thereby providing more amphipathic regions exposed to the oil–water interface, where adsorption occurs [75]. Improved water and oil retention capabilities have been reported for most proteins treated with MAE due to enhanced surface area and modified pore structure of the microwave-exposed protein particles [91]. On the other hand, excessively treated proteins tend to reaggregate, diminishing foamability and emulsion, thus necessitating careful determination of optimum power and time exposure [68].

Physiochemically, microwave irradiation-induced conformational modifications in almond proteins characterized by decreases in α-helix content and increases in β-sheet and random coil structures demonstrated partial protein unfolding without total denaturation at moderate power levels [75,86]. Bioactive peptides released from protein structures via microwave-induced unfolding displayed greatly enhanced antioxidant activities relative to those from conventional hydrolysis processes, as evidenced by ORAC values that were much higher [81,92]. Importantly, moderate-power microwave processing preserved the nutritional quality of almond proteins, as evidenced by similar essential amino acid (EAA) profiles in both samples and enhanced in vitro digestibility, facilitated by easier access of gastrointestinal proteolytic enzymes to peptide bonds [6,54].

### 6.3. High-Pressure Processing

High-pressure processing (HPP), a cold process, uses pressures (typically between 100 and 600 MPa) that result in alterations to proteins’ three-dimensional arrangement without the degradation associated with heat. As such, HPP breaks up some of the weaker intermolecular interactions such as hydrogen bonding, hydrophobic interaction, and electrostatic attraction whilst retaining the stronger peptide linkages, thus allowing for specific manipulation of protein unfolding/association/dissociation and rearrangement of secondary and tertiary structures [93,94]. Given the superior nutritional attributes of almond proteins, both high-pressure microfluidization (HPM) and high-pressure homogenization (HPH) represent possible approaches to the alteration of the molecular structure of almond proteins and subsequently their functional properties [83,95].

When HPH is applied to almond milk at pressures of 62–172 MPa the particles formed are all of similar size and exhibit a greatly reduced diameter when compared to those produced by conventional methods. The HPH method also resulted in an increase in the absolute zeta-potential value, suggesting improved electrostatic repulsive force between protein molecules, i.e., improved colloidal stability [96].

When almond protein dispersions were treated under conditions of high hydrostatic pressure (HHP) for periods of 5–20 min at 200–600 MPa, The degree of structural modification achieved was dependent upon both the pressure used and the duration for which the sample was held at this pressure [27,97].

HPP has been shown to significantly alter the techno-functional properties of almond proteins. Microfluidization at 160 MPa led to considerable reductions in mean particle diameter and increases in absolute zeta-potential values. It is suggested that this would enhance colloidal stability and electrostatic repulsion between protein molecules [83], potentially improving protein solubility. Hydrophobic amino acids become exposed during protein dissolution, thereby improving emulsification properties. Therefore, microfluidization treatments conducted at high pressures significantly improved both the solubility and emulsification capabilities of almond proteins. Similarly, Bernat et al. [96] demonstrated that HPH of almond milk at a pressure of 172 MPa converted bimodal, polydisperse particle distributions into monodisperse systems having improved clarity and physical stability without changing either viscosity or protein stability. Liu et al. [98] demonstrated that the water-holding capacity and oil-holding capacity of almond hull powder significantly increased after HPH at a pressure of 152 MPa, resulting in improved emulsification properties. Foaming capabilities are generally preserved or slightly enhanced with moderate levels of pressure, although very high levels of pressure can cause aggregation that diminishes interfacial function [77,99].

Physicochemical alterations induced by HPP in almond proteins vary depending on the processing method used. FTIR analysis indicated that microfluidization at a pressure of 160 MPa resulted in decreased β-sheets and α-helices increased from 58.1% to 61.7%. The data indicate that pressure treatment caused protein reorganization consistent with that observed for thermally denatured proteins, but differed from it [83].

Furthermore, HHP treatment has been reported to reduce the immunogenicity of almond proteins. Synergistic effects on epitope disruption have been reported for various combinations of pressure, temperature, and treatment times [27,98]. Additionally, HPH treatment of almond hull at a pressure of 152 MPa was reported to improve the antioxidant capacity of extracts, including 2,2-diphenyl-1-picrylhydrazyl (DPPH) free-radical scavenging activity and ferrous reducing power [98]. Therefore, high-pressure processing appears to be an attractive non-thermal method for altering almond protein structure to improve techno-functional properties and physicochemical characteristics. However, careful optimization of pressure intensity, hold time, and processing mode should occur prior to applying this method.

### 6.4. Pulsed Electric Field (PEF) Extraction

PEF extraction is a non-thermal method for treating foods using an electrical discharge (usually 0.1–80 kV/cm) applied briefly (e.g., microseconds–milliseconds) to create transient holes in cell membranes (electroporation), allowing nutrients to be released into the surrounding fluid without generating significant amounts of heat. The mechanisms underlying PEF-assisted extraction of plant proteins comprise two concurrent events: electroporation of cell membranes, which facilitates the mass transfer of intracellular proteins into the extraction solution; and direct electric-field effects on protein molecules, including polarization of peptide dipoles and conformational unfolding of protein structures [100,101]. Process variables critical to successful operation of PEF include: electric field strength (kV/cm); pulse duration (ms^−1^); total number of pulses; pulse repetition rate (Hz); and specific energy input (kJ/kg). Plant protein extraction is typically conducted at lower energy levels than those required to destroy microbes. Specifically, moderate electric field strengths (i.e., 1–10 kV/cm) with relatively low specific energies (i.e., 10–100 kJ/kg) are used for extracting plant proteins. Higher electric field strengths result in increased joule heating and potential electrochemical side reactions that can lead to undesirable aggregation and oxidation of plant proteins [102,103]. First, Salgado-Ramos et al. [104] applied PEF as a pretreatment to almond hull biomass (3 kV/cm field strength, 100 kJ/kg specific energy, 100 μs pulses, 2 Hz) followed by supercritical-CO_2_ extraction to recover lipids, carbohydrates, and antioxidants from the hull. Hull biomass is an outer-layer by-product and the study did not target kernel protein. Similarly, treatment of almond protein systems with PEF can disrupt rigid cotyledon cell walls and protein bodies, resulting in release of amandin and other storage proteins into the aqueous extraction medium [67,73].

PEF treatments produce substantial improvements in techno-functional properties of plant proteins related to almond protein systems. PEF treatment polarizes protein molecules and redistributes surface charges, increasing the net charge on the protein surface and promoting electrostatic repulsion between molecules. This effect can improve protein solubility, particularly at pH values away from the isoelectric point [101]. Foamability increases because PEF treatment allows rapid protein molecule migration toward air-water interfaces allowing rapid film development. Improved water and oil absorption capacities result from PEF-generated structural modifications creating porous surfaces that increase contact areas between proteins and liquids [8]. However, excessive PEF intensity can cause reaggregation and diminished foamability and foam stability [105].

Electrical treatments induce distinct physical chemical modifications to protein structures. Spectroscopic techniques, i.e., FTIR and CD, provide evidence that there is a decrease in α-helix structures and an increase in β-sheet and random coil structures resulting from partial protein unfolding during PEF treatment. Partial unfolding prevents complete denaturation of the protein molecules. Increased surface hydrophobicity due to exposure of previously buried hydrophobic regions occurs simultaneously with a negatively charged zeta potential resulting from conformational rearrangement of charged amino acid residues within the protein molecules. Greater electrostatic repulsion among the newly formed charges results in improved colloidal stability. An increase in free thiol SH-group content occurs subsequently to PEF treatment of plant proteins, demonstrating disruption of disulfide-mediated bonding between adjacent cysteine residues.

The use of PEF provides a novel, non-thermal, environmentally friendly means for preparing extracts rich in functional almond proteins. Careful optimization of various process parameters including electric field strength and pulse parameters will be essential to maximize desirable structural changes in plant proteins during extraction.

## 7. Hybrid and Integrated Extraction Approaches

Emerging technologies such as ultrasound-assisted extraction (UAE), microwave-assisted extraction (MAE), enzyme-assisted extraction (EAE), pulsed electric field (PEF), and high-pressure processing (HPP) have shown potential to improve recovery and quality of almond proteins. They have also shown some limitations on their own. For example, long-term use of UAE may lead to localized hot spots and protein coagulation; high power levels of MAE poses protein thermal degradation risk; EAE may result in over-hydrolysis if it is used exclusively; and PEF will generate unwanted side reactions via electrochemistry [100]. By combining several of these technologies, referred to as hybrid or multistage extraction, we can potentially take advantage of some of the unique characteristics associated with each of them, eliminate many of their disadvantages, and enhance the overall amount of recoverable protein, preserve structure, and create products with better functional and physicochemical properties [106,107,108].

Combining an initial ultrasonic pretreatment step with subsequent enzymatic hydrolysis is one of the most popular hybrid approaches currently being investigated for the recovery of almond protein. As a first step, ultrasound-produced cavitation generates acoustic shock waves capable of disrupting both the outer membrane and inner cytoplasmic membranes of plant cells and folding back proteins into their native, more open, conformation, thus increasing the number of exposed peptide bonds available for subsequent enzyme–substrate interactions during the hydrolysis process [109]. Studies show that when ground de-oiled almond meal was sonicated (40 kHz, 200 W for 50 min) and then subsequently treated with Alcalase hydrolyzing enzymes, the total protein recovered was approximately 1.16-fold greater than control groups receiving no ultrasonic treatment prior to enzymatic hydrolysis. Moreover, the degree of hydrolysis (DH) also increased to 10.95% from 5.76%. Furthermore, the molecular weights the peptides produced in this way were lower, exhibited improved TCA-solubility index values and displayed dramatically improved antioxidant activity relative to those produced using conventional hydrolysis processes. Cavitation induced by ultrasound causes shear forces, leading to structural pre-disruption and creating a more porous and accessible substrate matrix. Therefore, less enzyme is needed along with less processing time to obtain desired DH values. Thus, this method addresses a major limitation of enzyme-based extraction systems requiring longer incubation times and higher enzyme dosages to break down cell walls limiting access to substrates [110].

Pretreating almond meal with microwave energy before enzymatic hydrolysis provides additional advantages to the ultrasound–enzyme-based system. Using a microwave frequency of 160 W for 45 s prior to Alcalase treatment, protein recovery increased 1.18-fold and DH was 8.87% [81]. Microwaves rapidly heat the interior of plant cells, generating sufficient pressure inside plant cells to rupture the cell wall and unfold proteins, making cleavage sites more accessible to proteolytic enzymes. Sodium dodecyl sulfate–polyacrylamide gel electrophoresis (SDS–PAGE) analysis indicates that microwave-pretreated hydrolysates had fewer high-molecular-weight protein bands, demonstrating the extensive fragmentation of proteins like amandin subunits. Additionally, the antioxidant activity of microwave-pretreated hydrolysates is significantly higher than that of enzyme-only hydrolysates, indicating that microwave-induced changes in protein structural characteristics make more bioactive peptide sequences with radical-scavenging activity available [107]. Due to the relatively short pretreatment (in seconds vs. minutes), lower energy requirements per unit mass of product compared to ultrasound treatments, and ability to be employed in continuous flow operations, the microwave–enzyme sequential strategy appears especially beneficial for large-scale commercial applications.

When ultrasound and microwaves are applied separately or together (sequential or simultaneous) this approach is known as ultrasound-microwave assisted extraction. A recent study comparing UAE and MAE for okara derived from almond milk shows that each technology attacks different aspects of cellular material microwaves act thermally to rupture cells, while ultrasound mechanically disrupts cell walls, creating synergies when used together [90]. Bioactive peptides were found to be produced at higher yields in less time when enzymatically hydrolyzed under USMW conditions than with either technique applied separately. The mechanical vibrations created by ultrasound enhances uniform penetration of microwave energy across sample surfaces while eliminating temperature gradients across sample materials [107,111]. Pretreatment with both USMW and enzyme processing results in structural modification including increased surface area, broken polysaccharide linkages in cell-wall matrices, and partially unfolded protein conformations that facilitate more rapid and complete enzymatic digestion. Compared with other individual techniques, proteins extracted by the combined USMW process exhibit greatly increased solubility/emulsification properties, as well as enhanced antioxidant capacity [112].

Pulsed electric field (PEF) and ultrasound (US), when applied together to almond seeds, exhibit a highly effective synergy. The PEF-induced membrane electroporation creates pores in the cell walls, which allow the ultrasound-generated cavitation jet to enter the cells and break down the proteins. Therefore, the release of intracellular proteins and co-extractable bioactives is increased. In general, it was observed that when almond seeds were treated with a combination of PEF-US treatments, the greatest quantity of total phenols, flavonoids, condensed tannins, and anthocyanins was extracted. Also, greater antioxidant activity was measured using DPPH, redox potential, and metal-chelating assay than when these two treatments were used separately [112]. When PEF is integrated with enzymes, allowing the first PEF to create openings in cell walls followed by enzymatic hydrolysis, this approach allows enzymes to have better access to the intracellular protein storage compartments. This leads to fewer enzyme requirements, but similar or even higher degrees of hydrolysis [100,108].

HPM is combined with posttreatments such as ultrasonication or enzymatic hydrolysis, and the resulting pressure-induced structural opening of aggregates yields a substrate that is more readily functionalized. HPP–enzymic combinations have been studied for almond proteins to enhance digestibility and decrease allergenicity. It was found that the pressure-induced unfolding of proteins exposed internal epitopes/buried cleavage sites, making them available for both immunomodulation and enzyme access [94,97]. Furthermore, almond protein isolate (API) subjected to high-pressure microfluidization (HPM) at 160 MPa resulted in API with smaller particles, higher absolute zeta-potential values, and a modification of its secondary structure, with a reduction in beta-sheet content and an increase in α-helix content from 58.1% to 61.7% [83].

Table 3 compares the protein recovery, the advantages, and the limitations associated with the emerging extraction technologies for almond protein extraction.

## 8. Comparative Analysis of Technologies

**Figure 4:**
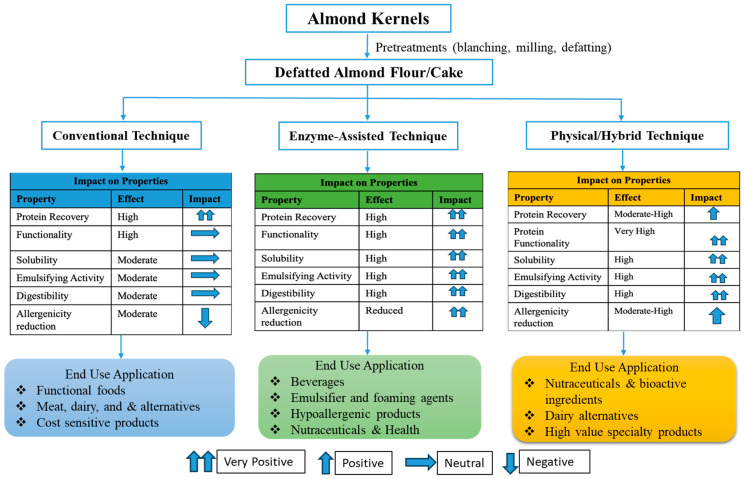
Comparative framework illustrating the effects of conventional, enzymatic, and physical/hybrid extraction technologies on almond protein recovery, functionality, solubility, emulsifying activity, digestibility, allergenicity, and end-use applications.

The increasing demand for plant-based protein ingredients has accelerated the development of efficient extraction technologies for almond proteins. While numerous studies have focused on optimizing individual extraction methods, direct comparisons reveal important trade-offs among protein recovery, preservation of functionality, process economics, scalability, and commercialization potential. Therefore, selecting an appropriate extraction strategy requires consideration of both technical performance and intended end-use applications. A comparative overview of the major extraction approaches and their impacts on almond protein properties and applications is presented in Figure 4.

From a protein recovery perspective, alkaline extraction–isoelectric precipitation (AE–IEP) and enzyme-assisted aqueous extraction (EAEP) generally provide the highest extraction efficiencies among currently available methods. AE–IEP remains widely utilized because of its simplicity, relatively low processing cost, and ability to produce highly purified protein isolates under industrially feasible conditions [43,44]. Similarly, EAEP enhances protein recovery by disrupting cell-wall polysaccharides and protein–matrix interactions through hydrolytic action, frequently achieving higher extraction yields than conventional aqueous extraction alone [11,14]. Hybrid approaches integrating ultrasound or microwave pretreatments with enzymatic hydrolysis have demonstrated further improvements in protein recovery by enhancing mass transfer and increasing enzyme accessibility to intracellular proteins [80,81].

However, maximizing extraction yield does not necessarily ensure preservation of protein functionality. The alkaline conditions employed during AE–IEP may induce protein denaturation, aggregation, and conformational changes that negatively affect solubility and certain interfacial properties [43,50]. In contrast, salt extraction preserves the native structure of amandin because extraction occurs under near-neutral pH conditions, resulting in protein with higher solubility, excellent functional properties, and structural integrity [25,26,61], but high salt content in the extract, requiring expensive desalting to obtain purified protein. Likewise, non-thermal emerging technologies such as ultrasound-assisted extraction (UAE), pulsed electric field (PEF), and high-pressure processing (HPP) primarily modify non-covalent interactions without extensive peptide bond cleavage, thereby maintaining desirable functional characteristics while improving extraction performance [83,93,98]. Enzyme-assisted extraction occupies an intermediate position because controlled hydrolysis can significantly improve solubility, digestibility, emulsification, and foaming properties; however, excessive hydrolysis may impair gelation behavior and reduce long-term emulsion stability [12,79,80].

Industrial feasibility and scalability represent critical considerations for commercial implementation. So far, AE–IEP is still the industry standard method for plant protein extraction because it utilizes well-established equipment, relatively simple processing steps, has the lowest production costs, and has extensive industrial use for oilseed and legume protein production [8,43]. Salt extraction provides excellent functional protein ingredients, but requires costly downstream desalting operations such as dialysis, ultrafiltration, or diafiltration, limiting large-scale adoption [58,60]. Enzyme-assisted extraction technologies are commercially attractive for producing premium protein ingredients with enhanced functionality and digestibility; however, enzyme costs, process control requirements, and longer processing times remain important constraints [75,80]. In contrast, emerging physical technologies including UAE, MAE, HPP, and PEF require substantial capital investment and specialized equipment, while industrial-scale validation remains relatively limited compared with conventional extraction approaches [68,75,96]. Consequently, comprehensive technoeconomic analyses and pilot-scale studies remain necessary before widespread commercialization of these technologies can occur.

The suitability of extraction technologies also varies according to the intended application of the final protein ingredient. For bulk protein isolates and food fortification applications, AE–IEP remains attractive because of its high protein purity and established industrial feasibility [43,44]. Beverage applications require highly soluble proteins with excellent dispersion stability, making salt extraction, EAEP, and mild physical extraction technologies particularly advantageous [14,26,61]. Proteins intended for dairy alternatives, emulsified products, and plant-based beverages benefit from extraction methods that preserve emulsification capacity and interfacial functionality, such as UAE, HPP, and controlled enzymatic treatments [83,96]. Furthermore, hypoallergenic formulations may benefit from controlled protease-assisted extraction because enzymatic hydrolysis can significantly reduce almond protein immunoreactivity through modification of allergenic epitopes [54]. Nutraceutical applications represent another promising area where enzyme-assisted and hybrid extraction technologies can generate bioactive peptides with antioxidant, α-glucosidase inhibitory, and health-promoting activities [45,53,54].

Overall, no single extraction technology can simultaneously maximize protein recovery, preserve native functionality, minimize production costs, ensure sustainability, and satisfy all end-use requirements. Conventional technologies currently offer superior scalability and commercialization readiness, whereas emerging and hybrid extraction approaches provide greater opportunities for producing high-functionality and value-added protein ingredients. Future industrial development will likely focus on integrated extraction platforms that combine the scalability of conventional processing with the functionality-preserving advantages of emerging technologies, thereby enabling the production of application-specific almond protein ingredients for diverse food and nutraceutical applications.

## 9. Conclusions

Almond nuts have a firmer and denser texture than other nuts. Although they contain a significant amount of protein (18–26%), they are also rich in lipid and coated with reddish brown skin rich in polyphenols. Efficient protein recovery from almond kernels remains technically challenging due to the dense cotyledon structure, high lipid content, strong protein–lipid interactions, and the presence of polyphenol-rich seed coats. Consequently, preprocessing operations such as blanching, grinding, and defatting play a critical role in determining extraction efficiency, structural integrity, and final functionality of almond protein ingredients.

Conventional alkaline extraction methods remain effective at achieving relatively high protein recovery, but they often compromise protein functionality through denaturation, aggregation, and chemical modification. Emerging approaches including enzyme-assisted, ultrasound-, microwave-, pulsed electric field-, and pressure-assisted extraction methods offer important advantages because they can improve mass transfer and preserve functional properties under milder processing conditions. Nevertheless, these technologies still face significant challenges related to processing cost, equipment requirements, scalability, process standardization, energy demand, and downstream separation efficiency.

## 10. Future Research Needs and Commercialization Challenges

Although considerable progress has been made in almond protein extraction and modification technologies, several scientific, technological, and commercial challenges must be addressed before these approaches can be widely adopted on an industrial scale. Most published studies have been conducted under laboratory conditions, with limited validation at pilot or commercial scales. Therefore, future studies should also focus on developing process–structure–function relationships for almond proteins and reducing the cost of effective extraction process. In particular, more research is needed to understand preprocessing conditions, extraction parameters, and protein structural modifications influence on solubility, emulsification, gelation, digestibility, flavor interactions, and allergenicity in final food systems. Comparative pilot-scale studies and life-cycle assessments are also required to determine the commercial feasibility of green extraction technologies. In addition, combining extraction technologies with membrane filtration, enzyme recycling, fermentation, or precision hydrolysis may offer new opportunities to produce high-functioning almond protein ingredients with improved sustainability and reduced environmental impact.

In addition to protein recovery and functional and bioactive property preservation, allergenicity remains an important consideration in almond protein extraction. Almond proteins are highly allergenic and can lead to severe allergic reactions including life-threatening anaphylaxis. The major allergenic protein amandin (Pru du 6) is stable against heat destruction. Standard water-based extraction methods typically preserve the conformational characteristics and structural integrity of almond allergens, yielding proteins with high antigenicity. Enzyme-assisted aqueous extraction (EAEP) using proteases is promising in reducing the allergenicity of almond protein extract, but the degree of hydrolysis has to be strictly controlled depending on the end use to reduce the negative impacts of enzyme hydrolysis on the functional properties of almond protein. Ultrasound-assisted extraction (UAE), high-pressure extraction (HPE), and pulsed electric field-assisted extraction (PEFAE) methods also exhibit certain potential to reduce the allergenicity of almond protein. Therefore, future research needs to address the optimization of these non-conventional extraction methods and hybrid strategies to reduce almond protein allergenicity while enhancing the protein recovery, bioactivity and techno-functional properties. Advanced proteomic and immunological techniques may provide deeper insights into the structural changes responsible for allergenicity reduction.

Further, the adoption of any novel extraction process in the almond processing industry must meet regulation requirements. Companies must strictly navigate regulations for allergen management, solvent/chemical usage, and raw material safety. Because almonds are a major food allergen, extraction requires rigorous allergen control plans, facility sanitation validation, and accurate labeling on finished products. If enzymes or chemicals are used for extraction, they must be generally recognized as safe (GRAS) or approved as a food additive in the United States, or be evaluated by the European Food Safety Authority (EFSA) to ensure they do not pose a safety risk. In addition, almonds are susceptible to mold growth and aflatoxin contamination, and the final protein fractions must be tested against global maximum-aflatoxin-residue limits. All these regulatory requirements often slow the commercialization of a new extraction technology because it will take a long time for a company to collect sufficient evidence to prove that the materials and process meet the requirements.

Consequently, the future of almond protein processing will likely depend on the development of integrated and application-specific extraction strategies that can balance protein recovery, functional quality, economic viability, and environmental impact. The optimization of extraction parameters and different hybrid approaches using artificial intelligence-assisted process optimization may hasten the development, scaling up, and applications of novel technologies. Such advances will support the broader industrial utilization of almond proteins in sustainable plant-based food systems.

## Figures and Tables

**Table 1:** Protein recovery and functional properties of almond protein extracted by different conventional extraction techniques.

Techniques	Optimal Extraction Condition	Protein Recovery/Yield	Functional Properties of Protein Extract	Reference
Alkaline Extraction–Isoelectric Precipitation (AE–IEP)	Extraction solvents: NaOH, buffered saline borate (BSB), and Tris–HCl protein isolation by AE–IEP	Protein yield with BSB: 56% Protein yield with Tris-HCl buffer: 37%	High surface hydrophobicity, WHC and OBC increased protein solubility Increased emulsion activity index at pH 4–4.5	[63]
	Protein extraction from defatted almond flour AE–IEP Functional characterization at pH 4 and pH 7	Protein content of API: 94 ± 1%, Protein yield: 23 ± 3% (dry basis)	Protein solubility: 70–80% at both pH 4 and pH 7 Increased random coil structure at pH 4 Improved foam formation and stability Form heating produced gels with distinct microstructures particulate gel at pH 7, dense and continuous gel at pH 4	[32]
Aqueous Extraction Process (AEP) and Enzyme-Assisted Aqueous Extraction Process (EAEP)	Full-fat almond flour AEP condition: pH 9.0, 50 °C, 1:10 solid-to-liquid ratio, 60 min EAEP condition: Same as AEP with addition of 0.5% protease	Protein content of dry extracts: AEP extract: 57.33 ± 0.18% EAEP extract: 59.27 ± 0.68%	EAEP resulted in smaller peptides, reduced surface hydrophobicity Enhanced solubility at pH 3–6 Improved WAC, emulsification capacity and foaming capacity	[14]
	Almond flour pH 6.5–9.5, temperature 45–55 °C, solids-to-liquid ratio (SLR) 1:12–1:8, enzyme concentration 0.5–1.0% Optimal extraction conditions: pH 9.0, 50 °C, 1:10 SLR, 0.5% enzyme, 2 h	Total protein yields under optimal conditions AEP: 69.6% EAEP: 77.7%	EAEP produced more soluble peptides, particularly at acidic pH (pH 5.0) EAEP improved protein solubility under acidic conditions	[11]
Salt Extraction Methods (Micellar/Buffer)	Defatted almond flour Extraction solvent: Buffered saline borate (BSB; 0.1 M H_3_BO_3_, 0.025 M Na_2_B_4_O_7_, 0.075 M NaCl, pH 8.45)	Protein yield of ~27.4% Protein content almond protein isolates: ~93%	Exhibited good aqueous solubility High viscosity, oil-holding capacity, & gelation ability	[63]

**Table 2:** Comparison of principles, advantages, and disadvantages of conventional extraction techniques.

Extraction Techniques	Principle	Advantages	Disadvantages	Reference
Alkaline Extraction–Isoelectric Precipitation (AE–IEP)	Raising the pH above the isoelectric point makes proteins negatively charged, increasing their solubility. Addition of acids in the extracted protein solution to the protein’s isoelectric point (pI), where net charge is zero, minimizes the protein solubility, causing precipitation.	Relatively simple and cost-effective processing. Moderate emulsifying capacity and emulsion stability. Produces highly purified amandin with high thermal stability. Structural resilience, and colloidal stability.	Poor solubility and foaming near pI. Potential Maillard browning under alkaline conditions. Phenolic-induced color and bitterness. Loss of albumin fraction during isoelectric precipitation.	[39,64]
Aqueous Extraction Process (AEP)	Protein aqueous extraction without alkaline addition relies purely on the natural solubility of proteins in water. This method uses mechanical and physical forces (homogenization) to disrupt cell structures to facilitate the release of proteins from cellular matrix.	Environmentally friendly process enabling simultaneous recovery of oil and protein. Improves protein extractability and forms stable protein. Stabilized emulsions. Avoids flammable solvents.	Produces stable cream emulsions. Requires demulsification steps to separate protein from lipid. Increases processing complexity, and energy demand. Potential protein structural modification.	[53,54]
Salt Extraction Methods (Micellar/Buffer)	Enhance globulin solubility through ionic strength effects through “salting in) effect at high salt concentration (NaBr saline buffer), followed by micellar precipitation (Salting Out) or centrifugation, and/or dialysis to desalt.	Efficiently solubilizes almond globulins including amandin. Maintaining structural integrity. Improves protein extractability.	Low extraction efficiency and analytical sensitivity. Yields low-purity crude extracts. Needs costly and complex downstream processing to desalt. Retain allergenicity of defatted almond protein.	[53,54,65,66]

**Table 3:** Comparison of protein recovery, advantages, and disadvantages of emerging extraction techniques.

Techniques	Protein Recovery	Advantages	Disadvantage	Reference
Enzyme-assisted aqueous extraction process (EAEP)	Cell wall degrading enzymes (CWDEs) break down polysaccharides of the cell walls, leading to protein release into the extraction media Proteolytic enzymes hydrolyze proteins into smaller peptides soluble in water	Relatively mild process conditions Dramatically increases total protein yield Reduce immunoreactivity of almond protein extract Improve digestibility EAEP is sustainable and can serves as a green alternative to harsh chemical treatments. Extracts exhibit greater bioactivity Improve functional properties of protein extract at low to moderate DH	Proteases and the necessary temperature-control equipment can increase cost Extensive hydrolysis can sometimes introduce bitter peptides Microbial growth: Prolonged incubation at warm temperatures may require additional sterilization controls Have negative effects on the functional properties of protein extract at high DH	[11,12,14,54,80]
Ultrasound-Assisted Extraction (UAE)	68–77% protein recovery varied with ultrasound intensity and treatment time Protein yield from almond cake was 49.3 g/100 g Increase protein solubility by 53%	High protein yield Shorter extraction time Improved foaming capacity and water absorption Operates in an energy-efficient manner Eco-friendly Suitable for obtaining protein ingredients for human use	Cavitation can generate free radicals Requires careful parameter control to avoid protein damage Excessive ultrasound intensity or prolonged exposure (>50 min) results in protein degradation and aggregation High equipment cost and scalability challenge	[81,113]
Microwave-Assisted Extraction (MAE)	65–75% protein recovery	Significantly higher extraction yield and protein recovery than conventional methods Drastically reduced extraction time from hours to minutes, saving time and energy	Rapid heating may lead to protein denaturation and aggregation if not optimized Potential uneven heating High equipment cost Expensive to scale up	[81]
High-Pressure Processing (HPP/HPM)		Enhance protein extraction efficiency Preserve protein structure, enhance functionality Effectively reduce immunoreactivity of almond proteins at pressure 500 MPa or higher Eco-friendly	Greatly reduced the solubility almond proteins High pressures are energy-intensive and require specialized equipment Scalability challenges	[84,93]
Pulsed Electric Field (PEF)		Enhanced release of almond bioactives (proteins and polyphenols) Induced structural modifications, Improved stability increased phenolics and antioxidant activity	PEF at low field strengths (<1 kV/cm) had limited impact, while extended treatment risked electrode corrosion contamination and protein-specific functional properties remained unassessed	[114]
Ultrasound–Enzyme Hybrid	68–77% protein recovery	Promising approach for valorization of almond meal by-product Improved functional properties High antioxidant activity and iron-chelating capacity	Equipment and enzyme costs remain a barrier to scale up. Potential of excessive hydrolysis leading to loss of functionality Long hydrolysis time may increase energy cost and risk of microbial contamination	[81,115]
Polyethylene glycol (PEG)—Microwave-Enzyme Hybrid	98.81% protein recovery	Drastically reduced extraction time from hours to minutes, saving time and energy Potential to be used for other plant proteins	Not a purely “green” solvent-free method PEG may interfere with downstream processing and product applications	[116]

## Data Availability

The original contributions presented in the study are included in the article. Further inquiries can be directed to the corresponding author.

## References

[1] Johnson A.J., Stevenson J., Pettit J., Jasthi B., Byhre T., Harnack L. (2025). Assessing the nutrient content of plant-based milk alternative products available in the United States. J. Acad. Nutr. Diet..

[2] Ramsing R., Santo R., Kim B.F., Altema-Johnson D., Wooden A., Chang K.B., Semba R.D., Love D.C. (2023). Dairy and plant-based milks: Implications for nutrition and planetary health. Curr. Environ. Health Rep..

[3] Xie A., Dong Y., Liu Z., Li Z., Shao J., Li M., Yue X. (2023). A review of plant-based drinks addressing nutrients, flavor, and processing technologies. Foods.

[4] AgFunderNews (2024). US Retail Sales of Plant-Based Milk by Numbers: Coconut Is Up, Almond Is Down, Soy and Oat Are Flat. https://agfundernews.com/us-retail-sales-of-plant-based-milk-by-numbers-coconut-is-up-almond-is-down-soy-and-oat-are-flat.

[5] Yada S., Lapsley K., Huang G. (2011). A review of composition studies of cultivated almonds: Macronutrients and micronutrients. J. Food Compos. Anal..

[6] Shi T., Cao J., Cao J., Zhu F., Cao F., Su E. (2023). Almond (*Amygdalus communis* L.) kernel protein: A review on the extraction, functional properties and nutritional value. Food Res. Int..

[7] Barreca D., Nabavi S.M., Sureda A., Rasekhian M., Raciti R., Sanches Silva A., Annunziata G., Arnone A., Tenore G.C., Süntar İ. (2020). Almonds (*Prunus dulcis* Mill. D. A. Webb): A source of nutrients and health-promoting compounds. Nutrients.

[8] Ravindran N., Singh S.K., Singha P. (2024). A comprehensive review on the recent trends in extractions, pretreatments and modifications of plant-based proteins. Food Res. Int..

[9] Yang J.S., Dias F.F., Pham T.T.K., Barile D., de Moura Bell J.M. (2024). A sequential fractionation approach to understanding the physicochemical and functional properties of aqueous and enzyme-assisted aqueous extracted black bean proteins. Food Hydrocoll..

[10] Dias F.F.G., de Almeida N.M., SP de Souza T., Taha A.Y., LN de Moura Bell J.M. (2020). Characterization and demulsification of the oil-rich emulsion from the aqueous extraction process of almond flour. Processes.

[11] de Souza T.S.P., Dias F.F.G., Koblitz M.G.B., de Moura Bell J.M.L.N. (2019). Aqueous and enzymatic extraction of oil and protein from almond cake: A comparative study. Processes.

[12] de Almeida N.M., FG Dias F., Rodrigues M.I., de Moura Bell J.M.L.N. (2019). Effects of processing conditions on the simultaneous extraction and distribution of oil and protein from almond flour. Processes.

[13] Kumar M., Tomar M., Potkule J., Verma R., Punia S., Mahapatra A., Belwal T., Dahuja A., Joshi S., Berwal M.K. (2021). Advances in the plant protein extraction: Mechanism and recommendations. Food Hydrocoll..

[14] Dias F.F.G., de Moura Bell J.M.L.N. (2022). Understanding the impact of enzyme-assisted aqueous extraction on the structural, physicochemical, and functional properties of protein extracts from full-fat almond flour. Food Hydrocoll..

[15] Jain I., Kaur R., Kumar A., Paul M., Singh N. (2024). Emerging protein sources and novel extraction techniques: A systematic review on sustainable approaches. Int. J. Food Sci. Technol..

[16] Hewage A., Olatunde O.O., Nimalaratne C., Malalgoda M., Aluko R.E., Bandara N. (2022). Novel extraction technologies for developing plant protein ingredients with improved functionality. Trends Food Sci. Technol..

[17] Dourado F., Barros A., Mota M., Coimbra M.A., Gama F.M. (2004). Anatomy and cell wall polysaccharides of almond (Prunus dulcis DA Webb) seeds. J. Agric. Food Chem..

[18] Roncero J.M., Álvarez-Ortí M., Pardo-Giménez A., Rabadán A., Pardo J.E. (2020). Review about non-lipid components and minor fat-soluble bioactive compounds of almond kernel. Foods.

[19] Trombetta D., Smeriglio A., Denaro M., Zagami R., Tomassetti M., Pilolli R., De Angelis E., Monaci L., Mandalari G. (2020). Understanding the fate of almond (*Prunus dulcis* (Mill.) DA Webb) oleosomes during simulated digestion. Nutrients.

[20] Kleuter M., Yu Y., Pancaldi F., Nagtzaam M., van der Goot A.J., Trindade L.M. (2024). Cell wall as a barrier for protein extraction from tomato leaves: A biochemical study. Plant Physiol. Biochem..

[21] Nikiforidis C.V. (2019). Structure and functions of oleosomes (oil bodies). Adv. Colloid Interface Sci..

[22] Bolling B.W., Dolnikowski G., Blumberg J.B., Chen C.Y.O. (2010). Polyphenol content and antioxidant activity of California almonds depend on cultivar and harvest year. Food Chem..

[23] Mandalari G., Tomaino A., Arcoraci T., Martorana M., Turco V.L., Cacciola F., Rich G.T., Bisignano C., Saija A., Dugo P. (2010). Characterization of polyphenols, lipids and dietary fibre from almond skins (*Amygdalus communis* L.). J. Food Compos. Anal..

[24] Ozdal T., Capanoglu E., Altay F. (2013). A review on protein–phenolic interactions and associated changes. Food Res. Int..

[25] Wolf W.J., Sathe S.K. (1998). Ultracentrifugal and polyacrylamide gel electrophoretic studies of extractability and stability of almond meal proteins. J. Sci. Food Agric..

[26] Sathe S.K., Teuber S.S., Roux K.H. (2002). Effects of food processing on the stability of food allergens. Biotechnol. Adv..

[27] Zhang Y., Zhang J., Sheng W., Wang S., Fu T.J. (2016). Effects of heat and high-pressure treatments on the solubility and immunoreactivity of almond proteins. Food Chem..

[28] WHO/IUIS (2025). Allergen Nomenclature. World Health Organization and International Union of Immunological Societies (WHO/IUIS) Allergen Nomenclature Sub-Committee. https://allergen.org.

[29] Ahrens S., Venkatachalam M., Mistry A.M., Lapsley K., Sathe S.K. (2005). Almond (*Prunus dulcis* L.) protein quality. Plant Foods Hum. Nutr..

[30] Boye J., Zare F., Pletch A. (2010). Pulse proteins: Processing, characterization, functional properties and applications in food and feed. Food Res. Int..

[31] Carbonaro M., Nucara A. (2010). Secondary structure of food proteins by Fourier transform spectroscopy in the mid-infrared region. Amino Acids.

[32] Devnani B., Ong L., Kentish S., Gras S.L. (2021). Structure and functionality of almond proteins as a function of pH. Food Struct..

[33] Mandalari G., Mackie A.R. (2018). Almond allergy: An overview on prevalence, thresholds, regulations and allergen detection. Nutrients.

[34] Zhang Y., Jin T. (2020). Almond allergens: Update and perspective on identification and characterization. J. Sci. Food Agric..

[35] Fisklements M., Barrett D.M. (2014). Kinetics of almond skin separation as a function of blanching time and temperature. J. Food Eng..

[36] Verhoeckx K.C.M., Vissers Y.M., Baumert J.L., Faludi R., Feys M., Flanagan S., Herouet-Guicheney C., Holzhauser T., Shimojo R., van der Bolt N. (2015). Food processing and allergenicity. Food Chem. Toxicol..

[37] Ingegneri M., Smeriglio A., Rando R., Gervasi T., Tamburello M.P., Ginestra G., La Camera E., Pennisi R., Sciortino M.T., Mandalari G. (2023). Composition and biological properties of blanched skin and blanch water belonging to three sicilian almond cultivars. Nutrients.

[38] Bezerra M., Ribeiro M., Igrejas G. (2021). An updated overview of almond allergens. Nutrients.

[39] Ribeiro F.P., Naito R.S., Dos Santos Galvão B., Júnior W.F., Toyonaga K.K., Branco I.G., Ferreira S., Malacrida C.R. (2025). Innovative optimized protein extraction from pequi almond cake: A comparison of ultrasound-assisted and alkaline extraction methods. Food Chem. X.

[40] Rosenthal A., Pyle D.L., Niranjan K. (1996). Aqueous and enzymatic processes for edible oil extraction. Enzym. Microb. Technol..

[41] L’hocine L., Boye J.I., Arcand Y. (2006). Composition and functional properties of soy protein isolates prepared using alternative defatting and extraction procedures. J. Food Sci..

[42] Hallstrom J.R., Dias F.F.G., Yang J.S., de Moura J.M.L.N. (2025). From solid dispersion to the simultaneous extraction of lipids and proteins: A bio-guided strategy to improve the nutritional and biological properties of almond milk. Future Foods.

[43] Hou F., Ding W., Qu W., Oladejo A.O., Xiong F., Zhang W., He R., Ma H. (2017). Alkali solution extraction of rice residue protein isolates: Influence of alkali concentration on protein functional, structural properties and lysinoalanine formation. Food Chem..

[44] Fang B., Chang L., Ohm J.B., Chen B., Rao J. (2023). Structural, functional properties, and volatile profile of hemp protein isolate as affected by extraction method: Alkaline extraction–isoelectric precipitation vs salt extraction. Food Chem..

[45] de Souza T.S., Dias F.F., Oliveira J.P.S., de Moura Bell J.M., Koblitz M.G.B. (2020). Biological properties of almond proteins produced by aqueous and enzyme-assisted aqueous extraction processes from almond cake. Sci. Rep..

[46] Roncero J.M., Álvarez-Ortí M., Pardo-Giménez A., Rabadán A., Pardo J.E. (2021). Influence of Pressure Extraction Systems on the Performance, Quality and Composition of Virgin Almond Oil and Defatted Flours. Foods.

[47] Thannhauser T.W., Konishi Y., Scheraga H.A. (1984). Sensitive quantitative analysis of disulfide bonds in polypeptides and proteins. Anal. Biochem..

[48] Matos J.D.S., Costa J.E.G., Krichanã D.R.G.C., Azevedo P.Z., Nascimento A.L.A.A., Stringheta P.C., Martins E., Campelo P.H. (2024). Nut proteins as plant-based ingredients: Emerging ingredients for the food industry. Processes.

[49] Devnani B., Ong L., Kentish S.E., Scales P.J., Gras S.L. (2022). Physicochemical and rheological properties of commercial almond-based yoghurt alternatives to dairy and soy yoghurts. Future Foods.

[50] Cruz-Solis I., Ibarra-Herrera C.C., Rocha-Pizaña M.D.R., Luna-Vital D. (2023). Alkaline extraction–isoelectric precipitation of plant proteins. Green Protein Processing Technologies from Plants: Novel Extraction and Purification Methods for Product Development.

[51] Hadidi M., Aghababaei F., McClements D.J. (2023). Enhanced alkaline extraction techniques for isolating and modifying plant-based proteins. Food Hydrocoll..

[52] Freitas P.A., Martín-Pérez L., Gil-Guillén I., González-Martínez C., Chiralt A. (2023). Subcritical water extraction for valorisation of almond skin from almond industrial processing. Foods.

[53] de Souza T.S., Dias F.F., Koblitz M.G.B., de Moura Bell J.M. (2020). Effects of enzymatic extraction of oil and protein from almond cake on the physicochemical and functional properties of protein extracts. Food Bioprod. Process..

[54] Dias F.F.G., Huang Y.P., Schauer J., Barile D., Van de Water J., de Moura J.M.L.N. (2023). Effects of protease-assisted aqueous extraction on almond protein profile, digestibility, and antigenicity. Curr. Res. Food Sci..

[55] Zhou H.X. (2005). Interactions of macromolecules with salt ions: An electrostatic theory for the Hofmeister effect. Proteins Struct. Funct. Bioinform..

[56] Sathe S.K., Venkatachalam M., Sharma G.M., Kshirsagar H.H., Teuber S.S., Roux K.H. (2009). Solubilization and electrophoretic characterization of select edible nut seed proteins. J. Agric. Food Chem..

[57] Li S., Chu S., Lu J., Wang P., Ma M. (2018). Molecular and structural properties of three major protein components from almond kernel. J. Food Process. Preserv..

[58] Yaputri B.P., Bu F., Ismail B.P. (2023). Salt solubilization coupled with membrane filtration-impact on the structure/function of chickpea compared to pea protein. Foods.

[59] Lam A.C.Y., Can Karaca A., Tyler R.T., Nickerson M.T. (2018). Pea protein isolates: Structure, extraction, and functionality. Food Rev. Int..

[60] Vojdani F., Hall G.M. (1996). Solubility. Methods of Testing Protein Functionality.

[61] Sathe S.K. (1992). Solubilization, electrophoretic characterization and in vitro digestibility of almond (*Prunus amygdalus*) proteins 1, 2. J. Food Biochem..

[62] Zhang Q., Zhang X., Feng Y., Shi F. (2017). Compositions and physicochemical properties of sweet almond isolate proteins. Sci. Agric. Sin..

[63] Amirshaghaghi Z., Rezaei K., Habibi Rezaei M. (2017). Characterization and functional properties of protein isolates from wild almond. J. Food Meas. Charact..

[64] Albillos S.M., Menhart N., Fu T.J. (2009). Structural stability of amandin, a major allergen from almond (*Prunus dulcis*), and its acidic and basic polypeptides. J. Agric. Food Chem..

[65] Dias F.F.G., Teixeira B.F., Taha A.Y., de Moura Bell J.M.L.N. (2025). Integrated impact of environmentally friendly extraction and recovery methods on almond oil quality: Insights from a lipidomic perspective. J. Am. Oil Chem. Soc..

[66] Su M., Liu C., Roux K.H., Gradziel T.M., Sathe S.K. (2017). Effects of processing and storage on almond (*Prunus dulcis* L.) amandin immunoreactivity. Food Res. Int..

[67] Akyüz A., Tekin İ., Aksoy Z., Ersus S. (2024). Plant protein resources, novel extraction and precipitation methods: A review. J. Food Process Eng..

[68] Tong S.C., Siow L.F., Tang T.K., Lee Y.Y. (2025). Effect of Enzyme-Assisted Extraction on Structural and Functional Properties of Palm Kernel Protein. J. Food Biochem..

[69] Yeasmin F., Prasad P., Sahu J.K. (2025). Utilization of discarded Phaseolus vulgaris L. seeds for protein extraction by enzymatic modification: Protein physicochemical, techno-functional, and bioactive Characteristics. Food Bioprocess Technol..

[70] Gouseti O., Larsen M.E., Amin A., Bakalis S., Petersen I.L., Lametsch R., Jensen P.E. (2023). Applications of enzyme technology to enhance transition to plant proteins: A review. Foods.

[71] Wang H., Meng F., Zhao Y., Liu Y., Chen J., Liu X. (2025). Enzyme-assisted extraction of proteins from radish leaf and their structure-function relationship in stabilizing high internal phase emulsions compared to soy protein and whey protein. Food Hydrocoll..

[72] Rommi K., Hakala T.K., Holopainen U., Nordlund E., Poutanen K., Lantto R. (2014). Effect of enzyme-aided cell wall disintegration on protein extractability from intact and dehulled rapeseed (*Brassica rapa* L. and *Brassica napus* L.) press cakes. J. Agric. Food Chem..

[73] Liu C., Hao L., Chen F., Yang C. (2020). Study on Extraction of Peanut Protein and Oil Bodies by Aqueous Enzymatic Extraction and Characterization of Protein. J. Chem..

[74] Chandran A.S., Kashyap P., Thakur M. (2024). Effect of extraction methods on functional properties of plant proteins: A review. EFood.

[75] Tian L., You X., Zhang S., Zhu Z., Yi J., Jin G. (2024). Enhancing functional properties and protein structure of almond protein isolate using high-power ultrasound treatment. Molecules.

[76] Das S., Nadar S.S., Rathod V.K. (2021). Integrated strategies for enzyme assisted extraction of bioactive molecules: A review. Int. J. Biol. Macromol..

[77] Furia K.A., Majzoobi M., Torley P.J., Farahnaky A. (2026). Innovative approaches in leaf protein extraction: Advancements, challenges, and applications in sustainable food formulation and design. Crit. Rev. Food Sci. Nutr..

[78] Liu Y., Angelov A., Übelacker M., Baudrexl M., Ludwig C., Rühmann B., Sieber V., Liebl W. (2024). Proteomic analysis of Viscozyme L and its major enzyme components for pectic substrate degradation. Int. J. Biol. Macromol..

[79] Zhao J., Bhandari B., Gaiani C., Prakash S. (2022). Altering almond protein function through partial enzymatic hydrolysis for creating gel structures in acidic environment. Curr. Res. Food Sci..

[80] Bao Z., Zhao Y., Wang X., Chi Y. (2017). Effects of degree of hydrolysis (DH) on the functional properties of egg yolk hydrolysate with alcalase. J. Food Sci. Technol..

[81] Sari T.P., Sirohi R., Tyagi P., Tiwari G., Pal J., Kunadia N.N., Verma K., Badgujar P.C., Pareek S. (2024). Protein hydrolysates prepared by Alcalase using ultrasound and microwave pretreated almond meal and their characterization. J. Food Sci. Technol..

[82] Rahman M.M., Lamsal B.P. (2021). Ultrasound-assisted extraction and modification of plant-based proteins: Impact on physicochemical, functional, and nutritional properties. Compr. Rev. Food Sci. Food Saf..

[83] Sari T.P., Dhamane A.H., Pawar K., Bajaj M., Badgujar P.C., Tarafdar A., Bodana V., Pareek S. (2024). High-pressure microfluidisation positively impacts structural properties and improves functional characteristics of almond proteins obtained from almond meal. Food Chem..

[84] Yang Q., Han P., Qi W., Shao Y., Zhang X., Wu F., Zhang Z. (2025). High internal phase Pickering emulsion by ultrasound-modified almond protein isolate particles as a new fat substitute to improve oxidative stability of pork sausages. Front. Sustain. Food Syst..

[85] Riquelme N., Díaz-Calderón P., Luarte A., Arancibia C. (2025). Effect of ultrasound time on structural and gelling properties of pea, lupin, and rice proteins. Gels.

[86] Vanga S.K., Wang J., Raghavan V. (2020). Effect of pulsed ultrasound, a green food processing technique, on the secondary structure and in-vitro digestibility of almond milk protein. Food Res. Int..

[87] Zhang T., Jiang B., Wang Z. (2022). Ultrasound-assisted alkaline extraction of almond protein: Structural characterization and emulsifying properties. Ultrason. Sonochem..

[88] Gohi B.F.C.A., Du J., Zeng H.Y., Cao X.J., Zou K.M. (2019). Microwave pretreatment and enzymolysis optimization of the Lotus seed protein. Bioengineering.

[89] Habinshuti I., Mu T.H., Zhang M. (2020). Ultrasound microwave-assisted enzymatic production and characterisation of antioxidant peptides from sweet potato protein. Ultrason. Sonochem..

[90] Taha M., Dimitrov K., Samaillie J., Blanchemain N., Riviere C. (2025). Comparative analysis of microwave and ultrasound extraction techniques for valorizing almond milk byproducts. LWT.

[91] Bernardi S., Lupatini-Menegotto A.L., Kalschne D.L., Moraes Flores É.L., Bittencourt P.R.S., Colla E., Canan C. (2021). Ultrasound: A suitable technology to improve the extraction and techno-functional properties of vegetable food proteins. Plant Foods Hum. Nutr..

[92] Harkat-Madouri L., Touati H., Boulekbache-Makhlouf L., Madani K., Haddadi-Guemghar H. (2025). Optimization of the microwave-assisted extraction of total phenolic compounds (TPCs) from almond skins through artificial neural networks (ANNs) and assessment of the antioxidant and antihyperglycemic activity of the extracts. J. Food Process. Preserv..

[93] Queirós R.P., Saraiva J.A., da Silva J.A.L. (2018). Tailoring structure and technological properties of plant proteins using high hydrostatic pressure. Crit. Rev. Food Sci. Nutr..

[94] Braspaiboon S., Laokuldilok T. (2024). High hydrostatic pressure: Influences on allergenicity, bioactivities, and structural and functional properties of proteins from diverse food sources. Foods.

[95] Yang J., Liu G., Zeng H., Chen L. (2018). Effects of high pressure homogenization on faba bean protein aggregation in relation to solubility and interfacial properties. Food Hydrocoll..

[96] Bernat N., Chàfer M., Chiralt A., González-Martínez C. (2015). Effect of high pressure homogenisation and heat treatment on physical properties and stability of almond and hazelnut milks. LWT–Food Sci. Technol..

[97] De Angelis E., Bavaro S.L., Forte G., Pilolli R., Monaci L. (2018). Heat and pressure treatments on almond protein stability and change in immunoreactivity after simulated human digestion. Nutrients.

[98] Liu H., An J., Jiang B., Deng S. (2023). Application of high-pressure homogenization to improve physicochemical and antioxidant properties of almond hulls. J. Food Process Eng..

[99] Navare S.S., Karwe M.V., Salvi D. (2023). Effect of high pressure processing on selected physicochemical and functional properties of yellow lentil protein concentrate. Food Chem. Adv..

[100] Marín-Sánchez J., Berzosa A., Álvarez I., Sánchez-Gimeno C., Raso J. (2024). Pulsed electric fields effects on proteins: Extraction, structural modification, and enhancing enzymatic activity. Bioelectricity.

[101] Giteru S.G., Oey I., Ali M.A. (2018). Feasibility of using pulsed electric fields to modify biomacromolecules: A review. Trends Food Sci. Technol..

[102] Raso J., Heinz V., Alvarez I., Toepfl S. (2022). Pulsed Electric Fields Technology for the Food Industry.

[103] Nowosad K., Sujka M., Pankiewicz U., Kowalski R. (2021). The application of PEF technology in food processing and human nutrition. J. Food Sci. Technol..

[104] Salgado-Ramos M., Martí-Quijal F.J., Huertas-Alonso A.J., Sánchez-Verdú M.P., Cravotto G., Moreno A., Barba F.J. (2023). Sequential extraction of almond hull biomass with pulsed electric fields (PEF) and supercritical CO_2_ for the recovery of lipids, carbohydrates and antioxidants. Food Bioprod. Process..

[105] Malik M.A., Sheikh M.A., Mir N.A. (2024). A review on pulsed electric field modification of proteins: Effect on the functional and structural properties. Food Biosci..

[106] Chemat F., Vian M.A., Fabiano-Tixier A.S., Nutrizio M., Jambrak A.R., Munekata P.E., Lorenzo J.M., Barba F.J., Binello A., Cravotto G. (2020). A review of sustainable and intensified techniques for extraction of food and natural products. Green Chem..

[107] Zhou S., Chen W., Fan K. (2024). Recent advances in combined ultrasound and microwave treatment for improving food processing efficiency and quality: A review. Food Biosci..

[108] Issa R., Issa S., Rajapakse H. (2025). Next-generation extraction technologies for plant proteins: Enhancing yield, functionality, and sustainability. J. Biol. Methods.

[109] Zhang J., Ji S., Liang J., Chen Y., Tang W., Lyu F. (2024). Research progress and latest application of ultrasonic treatment on protein extraction and modification. Int. J. Food Sci. Technol..

[110] Mu Z.X., Liu B.X., Lai S.D., Wang Z.Y., Lv X.H., Yang L., Zhang H. (2025). Ultrasound-assisted enzymatic extraction of Pinus pumila nut protein: Effects on yield, physicochemical and functional properties. Food Sci. Nutr..

[111] Muñoz-Almagro N., Morales-Soriano E., Villamiel M., Condezo-Hoyos L. (2021). Hybrid high-intensity ultrasound and microwave treatment: A review on its effect on quality and bioactivity of foods. Ultrason. Sonochem..

[112] Khadhraoui B., Ummat V., Tiwari B.K., Fabiano-Tixier A.S., Chemat F. (2021). Review of ultrasound combinations with hybrid and innovative techniques for extraction and processing of food and natural products. Ultrason. Sonochem..

[113] Aiello G., Xu R., Pugliese R., Bartolomei M., Li J., Bollati C., Rueller L., Robert J., Arnoldi A., Lammi C. (2022). Quality assessment of the protein ingredients recovered by ultrasound-assisted extraction from the press cakes of coconut and almond beverage preparation. Foods.

[114] Manzoor M.F., Zeng X.A., Rahaman A., Siddeeg A., Aadil R.M., Ahmed Z., Li J., Niu D. (2019). Combined impact of pulsed electric field and ultrasound on bioactive compounds and FT-IR analysis of almond extract. J. Food Sci. Technol..

[115] Moayer S., Ghorbani M., Sadeghi Mahoonak A., Kashani Nejad M., Raeisi M. (2025). Optimizing production of hydrolyzed protein of sweet almond meal by ultrasound pretreatment and alcalase enzyme. J. Food Sci. Technol..

[116] Ge X.L., Shi T., Wang H., Zhang J., Zhang Z.Q. (2016). Development of an aqueous polyethylene glycol-based extraction and recovery method for almond (*Prunus armeniaca* L.) protein. Food Anal. Methods.

